# Assessment of cardio-renal-hepatic function in patients with valvular heart disease: a multi-biomarker approach—the cardio-renal-hepatic score

**DOI:** 10.1186/s12916-023-02971-y

**Published:** 2023-07-17

**Authors:** Junxing Lv, Bin Zhang, Yunqing Ye, Zhe Li, Weiwei Wang, Qinghao Zhao, Qingrong Liu, Zhenyan Zhao, Haitong Zhang, Bincheng Wang, Zikai Yu, Zhenya Duan, Shuai Guo, Yanyan Zhao, Runlin Gao, Haiyan Xu, Yongjian Wu, for the CHINA-VHD collaborators

**Affiliations:** 1 Department of Cardiology, Fuwai Hospital, National Center for Cardiovascular Diseases, Chinese Academy of Medical Sciences and Peking Union Medical College, No.167 Beilishi Road, Xicheng District, Beijing, 100037 China; 2 Medical Research & Biometrics Center, Fuwai Hospital, National Center for Cardiovascular Diseases, Chinese Academy of Medical Sciences and Peking Union Medical College, Beijing, China

**Keywords:** Valvular heart disease, Cardio-renal-hepatic function, Mortality, Prognosis

## Abstract

**Background:**

Valvular heart disease (VHD) can cause damage to extra-cardiac organs, and lead to multi-organ dysfunction. However, little is known about the cardio-renal-hepatic co-dysfunction, as well as its prognostic implications in patients with VHD. The study sought to develop a multi-biomarker index to assess heart, kidney, and liver function in an integrative fashion, and investigate the prognostic role of cardio-renal-hepatic function in VHD.

**Methods:**

Using a large, contemporary, prospective cohort of 6004 patients with VHD, the study developed a multi-biomarker score for predicting all-cause mortality based on biomarkers reflecting heart, kidney, and liver function (N-terminal pro-B-type natriuretic peptide [NT-proBNP], creatinine, and albumin). The score was externally validated in another contemporary, prospective cohort of 3156 patients with VHD.

**Results:**

During a median follow up of 731 (704–748) days, 594 (9.9%) deaths occurred. Increasing levels of NT-proBNP, creatinine, and albumin were independently and monotonically associated with mortality, and a weighted multi-biomarker index, named the cardio-renal-hepatic (CRH) score, was developed based on Cox regression coefficients of these biomarkers. The CRH score was a strong and independent predictor of mortality, with 1-point increase carrying over two times of mortality risk (overall adjusted hazard ratio [95% confidence interval]: 2.095 [1.891–2.320], *P* < 0.001). The score provided complementary prognostic information beyond conventional risk factors (C index: 0.78 vs 0.81; overall net reclassification improvement index [95% confidence interval]: 0.255 [0.204–0.299]; likelihood ratio test *P* < 0.001), and was identified as the most important predictor of mortality by the proportion of explainable log-likelihood ratio χ^2^ statistics, the best subset analysis, as well as the random survival forest analysis in most types of VHD. The predictive performance of the score was also demonstrated in patients under conservative treatment, with normal left ventricular systolic function, or with primary VHD. It achieved satisfactory discrimination (C index: 0.78 and 0.72) and calibration in both derivation and validation cohorts.

**Conclusions:**

A multi-biomarker index was developed to assess cardio-renal-hepatic function in patients with VHD. The cardio-renal-hepatic co-dysfunction is a powerful predictor of mortality and should be considered in clinical management decisions.

**Supplementary Information:**

The online version contains supplementary material available at 10.1186/s12916-023-02971-y.

## Background

With the rapid growth of aging population worldwide, valvular heart disease (VHD) has become a global health burden [1, 2]. Despite the constant evolution of technology and concept in management of VHD, a considerable proportion of patients still suffer significantly impaired survival [3–7]. Due to the long natural history of chronic VHD, patients are often in a complicated overall condition at the time of clinical decompensation, with a high prevalence of multi-organ damage, such as liver and kidney impairments [8–13], besides cardiac remodeling and dysfunction. Although the mechanisms of renal and hepatic dysfunction in VHD appear to be multifaceted, the role of heart-kidney-liver cross-talk, which is also known as “cardiorenal syndrome” and “cardiohepatic interaction”, can not be overlooked in this setting [9–12, 14–17]. Indeed, accumulating evidence supports that VHD can induce a series of systemic consequences and cause damage to the structure and function of extra-cardiac organs [9, 11, 12, 14, 16–20]. Such systemic impact of valvular dysfunction not only significantly impairs patients’ survival and quality of life, but may drive valvular intervention into futility [21, 22]. However, the major concern in management of VHD, as well as the main interest of current clinical research, were largely confined to imaging-derived cardiac parameters, with limited literature and vague recommendations on the implications of heart-kidney-liver interactions for prognostic evaluation and therapeutic decision making [22, 23].

Biomarkers provide a rapid and user-friendly approach to assess function of organs. In routine clinical practice, biomarker-based evaluation complements imaging method to a large extent, since the latter is not always available and generally requires a higher cost. In patients with VHD, previous studies suggested that natriuretic peptides were useful biomarkers for assessing cardiac function and patient prognosis [24–27]. Additionally, renal and hepatic function biomarkers were also prognostically meaningful in various VHD [8, 10, 12, 15–18, 21, 28, 29]. A multi-biomarker approach integrating heart, kidney, and liver function markers may refine systemic evaluation and improve risk prediction of VHD.

Therefore, the present study aimed to develop a multi-biomarker index enabling integrative assessment of heart, kidney, and liver function, as well as investigating the prognostic implications of cardio-renal-hepatic function in patients with significant VHD.

## Methods

### Study population

**Fig. 1:**
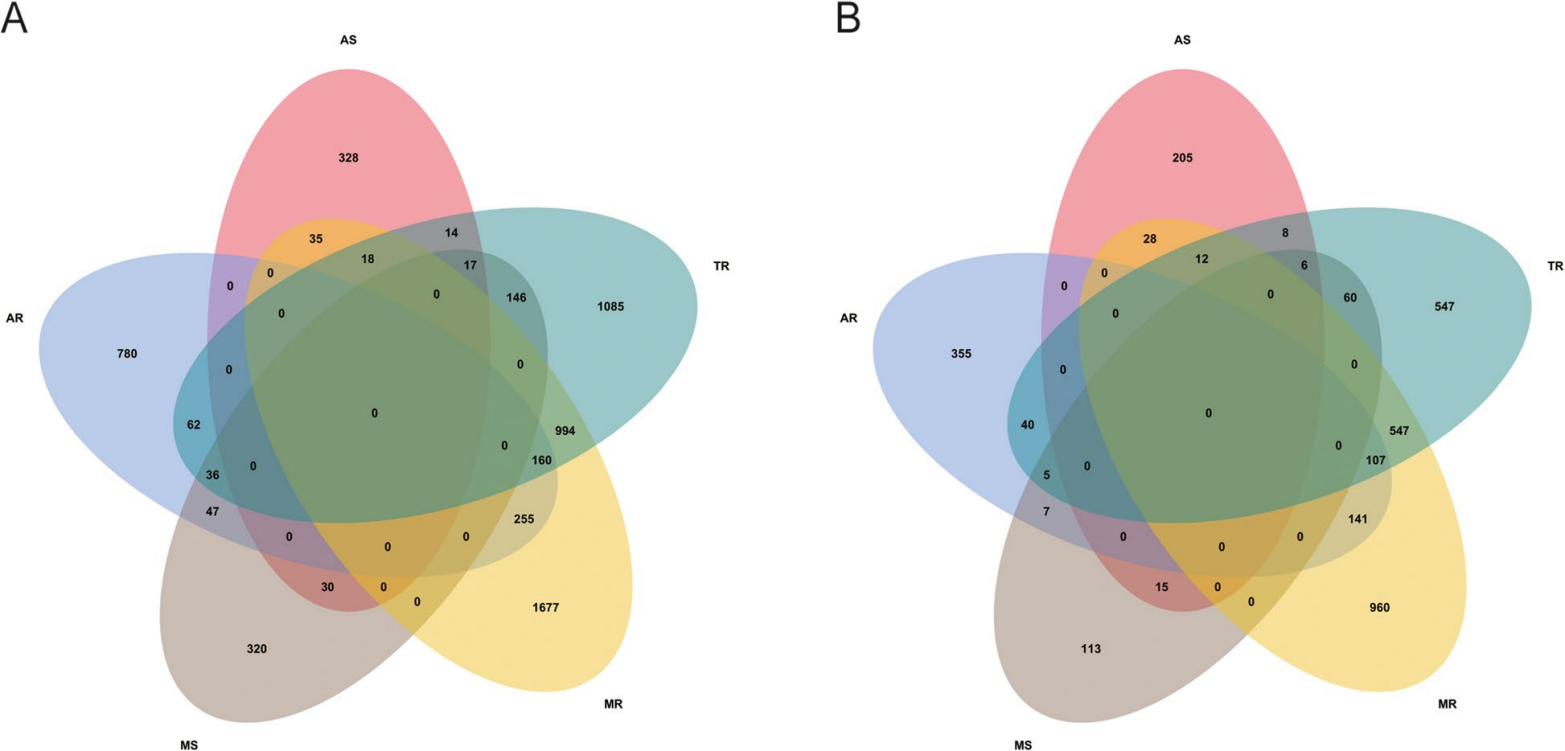
Venn diagrams on the distributions of VHD. **A** Venn diagram in the derivation cohort. **B** Venn diagram in the validation cohort. AS, aortic stenosis; AR, aortic regurgitation; MS, mitral stenosis; MR, mitral regurgitation; TR, tricuspid regurgitation; MVHD, multiple valvular heart disease; VHD, valvular heart disease

The China Valvular Heart Disease (China-VHD; NCT03484806) study was a nationwide, multicenter, prospective, observational study for adult patients (≥ 18 years) with significant VHD. Consecutive patients with at least moderate VHD, as identified by echocardiography, were enrolled between April and June 2018 from inpatient wards and outpatient clinics at 46 medical centers in China. Data collection and quality control of the China-VHD study have been described previously [17]. The study was performed in accordance with the Declaration of Helsinki and approved by the Institutional Review Board at Fuwai Hospital, National Center for Cardiovascular Diseases of China. Written informed consent was given by all eligible patients before registration. A total of 13,917 patients with various VHD were included in the China-VHD study. To conduct the present analysis, we excluded patients with moderate or greater tricuspid stenosis, pulmonary valve diseases, or mixed VHD. Patients with infective endocarditis, previous valvular interventions, the history of dialysis, missing value on heart, kidney, or liver biomarkers, as well as those without any follow-up information were also excluded. Finally, 6004 patients with aortic stenosis (AS), aortic regurgitation (AR), mitral stenosis (MS), mitral regurgitation (MR), tricuspid regurgitation (TR), and multiple valvular heart disease (MVHD) were included in the current study (Fig. 1; Additional file 1: Figure S1).

Details of the China Elderly Valve Disease (China-DVD; NCT02865798) study has been described previously [2, 24]. In brief, the China-DVD study was a nationwide, multicenter, prospective, observational study for elderly inpatients (≥ 60 years) with VHD. Inpatients with at least moderate VHD, as defined by echocardiography, were enrolled consecutively between September and December 2016 at 69 sites in China. The study protocol was approved by the central and site Institutional Review Board or Ethics Committees. Written informed consent was given by eligible patients before registration. Of 8929 patients enrolled in the China-DVD study, 3156 were included in the validation analysis (Fig. 1; Additional file 1: Figure S2).

### Echocardiography

In China-VHD study, comprehensive transthoracic two-dimensional and Doppler echocardiography was performed on all patients using standard ultrasound systems. The chamber quantification was performed based on the recommendations of American Society of Echocardiography and the European Association of Cardiovascular Imaging [30]. Left ventricular ejection fraction (LVEF) was calculated using the biplane modified Simpson method. Echocardiographic criteria of VHD were summarized in Additional file 2: Page S1. Quality control of echocardiography in the China-VHD study has been described previously [17]. The echocardiographic measurements, quality control, as well as echocardiographic criteria of significant VHD in the China-DVD study have been also described and published elsewhere [2].

### Biomarker measurement

Baseline venous blood samples were drawn after admission. Biomarker concentrations were determined during the same period of the baseline echocardiography. If there were multiple laboratory tests, the result of the first test after admission was collected in the databases. The plasma N-terminal pro-B-type natriuretic peptide (NT-proBNP) concentrations were measured using four assays, including Roche NT-proBNP Elecsys (Roche Diagnostics, Basel, Switzerland), Ortho Clinical Diagnostics Vitros ECi (Ortho Clinical Diagnostics, Raritan, New Jersey), BioMérieux NT-proBNP Vidas (Bio-Mérieux, Marcy, France), and Radiometer AQT90 Flex (Radiometer Medical Aps, Copenhagen, Denmark). The same antibodies and calibrator from the same vendor (Roche Diagnostics) were used by four assays.

### Outcomes

The primary outcome of the present study was all-cause mortality. When evaluating outcome of patients under conservative treatment, follow up was censored at the time of valvular intervention if performed.

### Statistical analysis

Data were presented as mean ± standard deviation (SD) or medians (interquartile range [IQR]) for continuous variables, and as counts (percentages) for categorical variables. Differences among groups were compared using Kruskal–Wallis test or Mann–Whitney U-test according to number of groups for continuous variables, and using Chi-square or the Fisher’s exact test for categorical variables as appropriate. The associations between the score and other variables were analyzed using Spearman correlation test and multiple linear regression models.

#### Associations of biomarkers with mortality

NT-proBNP and creatinine were selected as the components reflecting heart and kidney function in the cardio-renal-hepatic function index. Due to the skewed distribution of NT-proBNP and creatinine, the log_e_-transformations of two variables were used for analyses. To determine an appropriate index evaluating liver function, we compared the predictive performance of several hepatic biomarkers in overall population of the derivation cohort as well as in patients with various VHD using C index. Given the better predictive performance of albumin, a marker of liver synthesis, compared with other variables in total cohort and most types of VHD (Additional file 1: Table S1), it was selected to develop the multi-biomarker index.

Restricted cubic splines with 5 knots at 5th, 27.5th, 50th, 72.5th, and 95th percentiles were used to examine the shape of the associations of selected cardiac, renal, and hepatic biomarkers with mortality. Univariable and multivariable Cox proportional hazards models were also performed to analyze the associations between biomarkers and mortality, and to calculate hazard ratios (HRs) with 95% confidence intervals (CIs). Multivariable Cox models were adjusted for the following variables: age, sex, body mass index (BMI), smoking status, hypertension, hyperlipidemia, diabetes, prior myocardial infarction, cardiomyopathy, atrial fibrillation or flutter, chronic lung disease, New York Heart Association (NYHA) functional class (I-II/III-IV), hemoglobin, left atrial end-diastolic dimension (LA), left ventricular end-diastolic dimension (LVEDD), LVEF, pulmonary hypertension, severity of VHD, and valvular intervention. A minimally adjusted model incorporating age and sex was used in patients with MS due to a relatively small sample size. The proportional hazards assumptions were tested by the examination of log–log survival plots for categorical variables and Schoenfeld residual plots for continuous variables.

#### Development and validation of the cardio-renal-hepatic function index

NT-proBNP, creatinine, and albumin were fitted into a Cox regression model. A cardio-renal-hepatic function index, named the cardio-renal-hepatic (CRH) score, was developed based on these three biomarkers weighted by their regression coefficients. The CRH score was externally validated in the China-DVD cohort. C index was used to evaluate the performance of the score discrimination. The calibration property of the score was graphically examined by calibration curves presenting the association between observed and predicted survival probabilities.

#### Prognostic value of the CRH score in patients with VHD

We evaluated the prognostic value of CRH score as a continuous variable as well as categorically by quartiles. The Kaplan–Meier survival analysis was used to estimate the cumulative survival rates, and the differences among groups were compared by the log-rank test. Univariable and multivariable Cox proportional hazards models were performed to analyze the associations of the CRH score with mortality in both derivation and validation cohorts. The multivariable models were adjusted by covariates mentioned before. Relative importance of the CRH score compared with other variables was evaluated by the proportion of explainable log-likelihood ratio χ^2^ statistics, the best subset analysis, as well as the random survival forest analysis (Additional file 2: Page S1).

To analyze the incremental value of the CRH score beyond conventional risk factors, we assessed the potential improvement of predictive performance after including the CRH score into a base prognostic model, which was formed by all covariates mentioned before. The comparisons between models were performed using C index, net reclassification improvement index (NRI), integrated discrimination improvement index (IDI), likelihood ratio test, and Bayesian Information Criterion (BIC). The additional value of the score in term of clinical utility was examined by decision curve analysis.

A two-tailed *P* < 0.05 was considered to be statistically significant. Numbers of missing data and corresponding dispositions were summarized in Additional file 1: Table S2 and S3. All analyses in the present study were conducted using R version 4.2.2 (R Foundation for Statistical Computing, Vienna, Austria).

## Results

### Baseline characteristics

A total of 6004 patients with as least moderate VHD from the China-VHD study were included in the derivation cohort, and 3156 patients from China-DVD study were included in the validation cohort. The mean ages of two cohorts were 62.07 ± 13.80 and 71.24 ± 7.62 years, respectively. Baseline characteristics were summarized in Tables 1 and 2. The concentrations of NT-proBNP, creatinine, and albumin were 1447.22 pg/ml (468.00–3783.28 pg/ml), 82.44 μmol/L (69.00–100.00 μmol/L), and 3.94 ± 0.51 g/dL in the derivation cohort, and 1829.00 pg/ml (630.26–4510.07 pg/ml), 83.33 μmol/L (69.00–104.00 μmol/L), and 3.90 ± 0.62 g/dL in the validation cohort.

**Table 1:** Baseline characteristics

Variables	China-VHD (*n* = 6004)	China-DVD (*n* = 3156)
Age, yrs	62.07 ± 13.80	71.24 ± 7.62
Male sex	3413 (56.8)	1720 (54.5)
BMI, kg/m^2^	23.56 ± 3.69	23.32 ± 3.43
Current smoker	985 (16.4)	412 (13.1)
Hypertension	2657 (44.3)	1768 (56.0)
Hyperlipidemia	825 (13.7)	261 (8.3)
Diabetes	927 (15.4)	631 (20.0)
Coronary artery disease	2197 (36.6)	1405 (44.5)
Prior MI	679 (11.3)	376 (11.9)
Prior PCI	847 (14.1)	377 (11.9)
Prior CABG	159 (2.6)	77 (2.4)
Cardiomyopathy	621 (10.3)	298 (9.4)
Atrial fibrillation or flutter	1800 (30.0)	1321 (41.9)
Chronic lung disease	427 (7.1)	183 (5.8)
NYHA functional class		
I	1768 (29.4)	648 (20.7)
II	1364 (22.7)	762 (24.3)
III	2031 (33.8)	1182 (37.7)
IV	841 (14.0)	546 (17.4)
NT-proBNP, pg/ml	1447.22 (468.00–3783.28)	1829.00 (630.26–4510.07)
ln(NT-proBNP)	7.14 ± 1.59	7.38 ± 1.52
Hemoglobin, g/L	132.66 ± 20.78	127.03 ± 22.07
Creatinine, μmol/L	82.44 (69.00–100.00)	83.33 (69.00–104.00)
ln(Creatinine)	4.44 ± 0.34	4.46 ± 0.40
Albumin, g/dl	3.94 ± 0.51	3.90 ± 0.62
ALT, U/L	19.00 (13.00–30.68)	19.00 (13.00–30.00)
Total bilirubin, mg/dl	0.86 (0.61–1.25)	0.85 (0.61–1.24)
Direct bilirubin, mg/dl	0.27 (0.18–0.43)	0.26 (0.17–0.39)
MELD-XI score	10.21 (9.44–12.41)	10.45 (9.44–12.96)
LA, mm	45.82 ± 9.71	45.71 ± 9.12
LVEDD, mm	55.09 ± 11.35	55.18 ± 10.84
LVEF, %	56 (42–62)	54.2 (41–62.3)
Pulmonary hypertension	2562 (42.7)	1375 (43.6)
≥ moderate isolated AS	328 (5.5)	205 (6.5)
≥ moderate isolated AR	780 (13.0)	355 (11.2)
≥ moderate isolated MS	320 (5.3)	113 (3.6)
≥ moderate isolated MR	1677 (27.9)	960 (30.4)
≥ moderate isolated TR	1085 (18.1)	547 (17.3)
≥ moderate MVHD	1814 (30.2)	976 (30.9)
Valvular interventions	1922 (32.0)	674 (21.4)
Etiology		
Primary	3148 (54.7)	1712 (59.3)
Rheumatic	934 (29.7)	339 (19.8)
Degenerative	1541 (49.0)	1220 (71.3)
Congenital	479 (15.2)	113 (6.6)
Others	194 (6.2)	40 (2.3)
Secondary	2607 (45.3)	1177 (40.7)
Ischemic	586 (22.5)	387 (32.9)
Functional	2011 (77.1)	785 (66.7)
Others	10 (0.4)	5 (0.4)
Medication use		
Diuretics	4741 (79.0)	2396 (75.9)
Beta-blockers	3585 (59.7)	1950 (61.8)
ACEI/ARB	2436 (40.6)	1525 (48.3)
Warfarin	2282 (38.0)	1000 (31.7)
Aspirin	2203 (36.7)	1409 (45.7)
P2Y_12_ inhibitors	1522 (25.3)	872 (28.3)

Values are presented as mean ± standard deviation, median (interquartile range), or number (%). Baseline characteristics are shown before imputation of missing data

*VHD* valvular heart disease, *China-DVD* China Elderly Valve Disease, *BMI* body mass index, *MI* myocardial infarction, *PCI* percutaneous coronary intervention, *CABG* coronary artery bypass graft, *NYHA* New York Heart Association, *NT-proBNP* N-terminal pro-B-type natriuretic peptide, *ALT* alanine aminotransferase, *MELD-XI* Model for End-stage Liver Disease excluding international normalized ratio, *LA* left atrial end-diastolic dimension, *LVEDD* left ventricular end-diastolic dimension, *LVEF* left ventricular ejection fraction, *AS* aortic stenosis, *AR* aortic regurgitation, *MS* mitral stenosis, *MR* mitral regurgitation, *TR* tricuspid regurgitation, *MVHD* multiple valvular heart disease, *ACEI* angiotensin converting enzyme inhibitors, *ARB* angiotensin receptor blocker

**Table 2:** Baseline characteristics stratified by types of VHD

Variables	AS (*n* = 328)	AR (*n* = 780)	MS (*n* = 320)	MR (*n* = 1677)	TR (*n* = 1085)	MVHD (*n* = 1814)	*P* Value
Age, yrs	64.38 ± 11.32	60.54 ± 14.23	55.81 ± 11.09	61.38 ± 12.65	62.75 ± 16.81	63.64 ± 13.02	< 0.001
Male sex	196 (59.8)	606 (77.7)	96 (30.0)	1029 (61.4)	515 (47.5)	971 (53.5)	< 0.001
BMI, kg/m2	24.06 ± 3.31	24.00 ± 3.39	23.44 ± 3.28	23.98 ± 3.64	23.19 ± 4.13	23.15 ± 3.64	< 0.001
Current smoker	62 (18.9)	173 (22.2)	26 (8.1)	366 (21.8)	122 (11.2)	236 (13.0)	< 0.001
Hypertension	138 (42.1)	442 (56.7)	63 (19.7)	793 (47.3)	463 (42.7)	758 (41.8)	< 0.001
Hyperlipidemia	79 (24.1)	143 (18.3)	46 (14.4)	273 (16.3)	107 (9.9)	177 (9.8)	< 0.001
Diabetes	45 (13.7)	77 (9.9)	23 (7.2)	327 (19.5)	166 (15.3)	289 (15.9)	< 0.001
Coronary artery disease	116 (35.4)	294 (37.7)	48 (15)	791 (47.2)	342 (31.5)	606 (33.4)	< 0.001
Prior MI	18 (5.5)	64 (8.2)	6 (1.9)	313 (18.7)	98 (9.0)	180 (9.9)	< 0.001
Prior PCI	26 (7.9)	110 (14.1)	11 (3.4)	375 (22.4)	131 (12.1)	194 (10.7)	< 0.001
Prior CABG	10 (3.0)	17 (2.2)	6 (1.9)	60 (3.6)	31 (2.9)	35 (1.9)	0.050
Cardiomyopathy	4 (1.2)	28 (3.6)	0 (0.0)	287 (17.1)	79 (7.3)	223 (12.3)	< 0.001
Atrial fibrillation or flutter	21 (6.4)	72 (9.2)	145 (45.3)	367 (21.9)	408 (37.6)	787 (43.4)	< 0.001
Chronic lung disease	16 (4.9)	54 (6.9)	6 (1.9)	93 (5.5)	130 (12.0)	128 (7.1)	< 0.001
NYHA functional class							
I	83 (25.3)	300 (38.5)	80 (25.0)	493 (29.4)	440 (40.6)	372 (20.5)	< 0.001
II	85 (25.9)	258 (33.1)	106 (33.1)	363 (21.6)	203 (18.7)	349 (19.2)	
III	141 (43.0)	177 (22.7)	120 (37.5)	558 (33.3)	302 (27.8)	733 (40.4)	
IV	19 (5.8)	45 (5.8)	14 (4.4)	263 (15.7)	140 (12.9)	360 (19.8)	
NT-proBNP, pg/ml	891.55 (276.05–2670.50)	508.75 (135.85–1925.00)	646.05 (301.68–1251.75)	1480.00 (486.10–3795.50)	1331.00 (474.10–3425.75)	2519.50 (1091.00–5825.75)	< 0.001
ln(NT-proBNP)	6.74 ± 1.60	6.25 ± 1.76	6.42 ± 1.13	7.13 ± 1.61	7.09 ± 1.48	7.76 ± 1.33	< 0.001
Hemoglobin, g/L	133.37 ± 18.18	136.18 ± 18.73	134.34 ± 16.49	133.00 ± 20.09	133.67 ± 24.94	129.80 ± 20.28	< 0.001
Creatinine, μmol/L	76.00 (66.00–91.00)	83.95 (72.00–98.00)	75.00 (66.00–90.00)	84.11 (70.00–102.00)	80.80 (66.00–98.00)	84.00 (70.00–103.00)	< 0.001
ln(Creatinine)	4.36 ± 0.27	4.45 ± 0.30	4.34 ± 0.24	4.46 ± 0.35	4.41 ± 0.36	4.48 ± 0.37	< 0.001
Albumin, g/dl	4.11 ± 0.53	4.03 ± 0.49	4.18 ± 0.46	3.93 ± 0.51	3.91 ± 0.51	3.85 ± 0.50	< 0.001
ALT, U/L	16.30 (12.00–24.00)	17.00 (11.73–26.00)	19.00 (14.00–27.00)	20.60 (14.00–33.0)	18.80 (13.00–30.00)	20.00 (14.00–33.00)	< 0.001
Total bilirubin, mg/dl	0.68 (0.51–0.92)	0.73 (0.56–0.99)	0.78 (0.56–1.09)	0.81 (0.58–1.16)	0.92 (0.66–1.36)	1.00 (0.71–1.47)	< 0.001
Direct bilirubin, mg/dl	0.22 (0.15–0.30)	0.23 (0.16–0.32)	0.25 (0.17–0.33)	0.25 (0.17–0.39)	0.31 (0.20–0.49)	0.33 (0.22–0.54)	< 0.001
MELD-XI score	9.44 (9.44–10.67)	9.65 (9.44–11.49)	9.58 (9.44–11.13)	10.21 (9.44–12.22)	10.40 (9.44–12.82)	10.87 (9.44–13.45)	< 0.001
LA, mm	39.93 ± 6.34	39.88 ± 6.42	51.25 ± 9.55	46.65 ± 8.55	41.00 ± 8.45	50.60 ± 9.85	< 0.001
LVEDD, mm	50.12 ± 7.36	60.42 ± 10.35	46.86 ± 5.05	59.04 ± 10.06	45.89 ± 8.88	57.01 ± 11.31	< 0.001
LVEF, %	61 (55–66)	58 (50–62)	61 (56–65)	50 (35–60.7)	59 (51–63)	52 (38–60)	< 0.001
Pulmonary hypertension	38 (11.6)	63 (8.1)	127 (39.7)	492 (29.3)	673 (62.0)	1169 (64.4)	< 0.001
Severe VHD	252 (76.8)	289 (37.1)	179 (55.9)	525 (31.3)	347 (32.0)	1101 (60.7)	< 0.001
Valvular interventions	227 (69.2)	391 (50.1)	251 (78.4)	402 (24.0)	108 (10.0)	543 (29.9)	< 0.001
Etiology							
Primary	328 (100.0)	547 (73.8)	320 (100.0)	639 (39.1)	355 (37.2)	959 (54.0)	< 0.001
Rheumatic	35 (10.7)	47 (8.6)	306 (95.6)	120 (18.8)	32 (9.0)	394 (41.1)	
Degenerative	200 (61.0)	376 (68.7)	12 (3.8)	346 (54.1)	182 (51.3)	425 (44.3)	
Congenital	87 (26.5)	100 (18.3)	1 (0.3)	78 (12.2)	129 (36.3)	84 (8.8)	
Others	6 (1.8)	24 (4.4)	1 (0.3)	95 (14.9)	12 (3.4)	56 (5.8)	
Secondary	0 (0.0)	194 (26.2)	0 (0.0)	997 (60.9)	600 (62.8)	816 (46.0)	
Ischemic	0 (0.0)	0 (0.0)	0 (0.0)	382 (38.3)	0 (0.0)	204 (25.0)	
Functional	0 (0.0)	194 (100.0)	0 (0.0)	608 (61.0)	599 (99.8)	610 (74.8)	
Others	0 (0.0)	0 (0.0)	0 (0.0)	7 (0.7)	1 (0.2)	2 (0.2)	
Medication use							
Diuretics	249 (75.9)	592 (75.9)	275 (85.9)	1308 (78.0)	748 (68.9)	1569 (86.5)	< 0.001
Beta-blockers	209 (63.7)	477 (61.2)	153 (47.8)	1117 (66.6)	551 (50.8)	1078 (59.4)	< 0.001
ACEI/ARB	58 (17.7)	325 (41.7)	51 (15.9)	821 (49.0)	419 (38.6)	762 (42.0)	< 0.001
Warfarin	192 (58.5)	378 (48.5)	268 (83.8)	448 (26.7)	250 (23.0)	746 (41.1)	< 0.001
Aspirin	114 (34.8)	313 (40.1)	48 (15.0)	820 (48.9)	343 (31.6)	565 (31.1)	< 0.001
P2Y_12_ inhibitors	82 (25.0)	199 (25.5)	22 (6.9)	589 (35.1)	238 (21.9)	392 (21.6)	< 0.001

Values are presented as mean ± standard deviation, median (interquartile range), or number (%). Baseline characteristics are shown before imputation of missing data

*VHD* valvular heart disease, *AS* aortic stenosis, *AR* aortic regurgitation, *MS* mitral stenosis, *MR* mitral regurgitation, *TR* tricuspid regurgitation, *MVHD* multiple valvular heart disease, *BMI* body mass index, *MI* myocardial infarction, *PCI* percutaneous coronary intervention, *CABG* coronary artery bypass graft, *NYHA* New York Heart Association, *NT-proBNP* N-terminal pro-B-type natriuretic peptide, *ALT* alanine aminotransferase, *MELD-XI* Model for End-stage Liver Disease with albumin replacing international normalized ratio, *LA* left atrial end-diastolic dimension, *LVEDD* left ventricular end-diastolic dimension, *LVEF* left ventricular ejection fraction, *ACEI* angiotensin converting enzyme inhibitors, *ARB* angiotensin receptor blocker

### Development of the CRH score

**Fig. 2:**
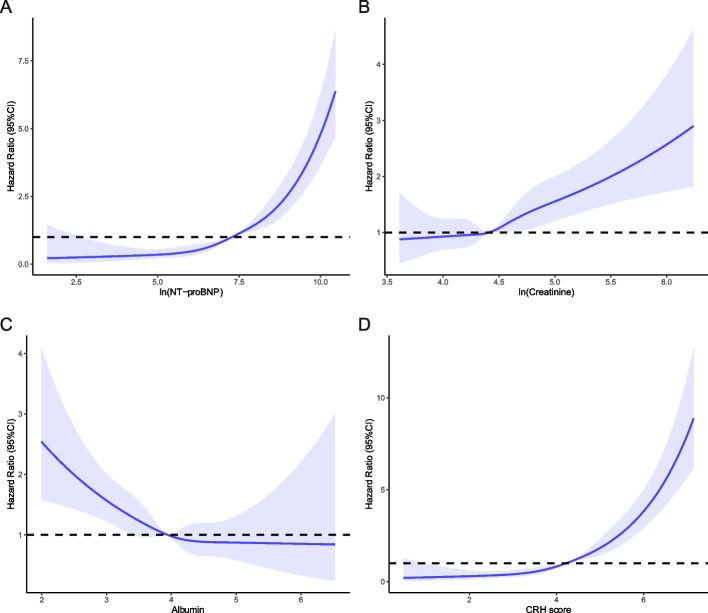
Restricted cubic splines for the associations of biomarkers with mortality. **A** The restricted cubic spline for the association of NT-proBNP with mortality. **B** The restricted cubic spline for the association of creatinine with mortality. **C** The restricted cubic spline for the association of albumin with mortality. **D** The restricted cubic spline for the association of CRH score with mortality. Adjusted for age, sex, BMI, smoking status, hypertension, hyperlipidemia, diabetes, prior myocardial infarction, cardiomyopathy, atrial fibrillation or flutter, chronic lung disease, NYHA functional class, hemoglobin, LA, LVEDD, LVEF, pulmonary hypertension, severity of VHD, and valvular intervention. The corresponding mortality risks to the median values of biomarkers were chosen as references. NT-proBNP, N-terminal pro-B-type natriuretic peptide; CRH, cardio-renal-hepatic; BMI, body mass index; NYHA, New York Heart Association; LA, left atrial end-diastolic dimension; LVEDD, left ventricular end-diastolic dimension; LVEF, left ventricular ejection fraction; VHD, valvular heart disease; CI, confidence interval

During a median follow up of 731 (704–748) days, 594 (9.9%) patients died in the China-VHD cohort. The cumulative survival at one and two years was 92.7% and 89.7%, respectively. The increasing levels of NT-proBNP, creatinine, and albumin were independently and monotonically associated with two-year mortality (Fig. 2; Additional file 1: Table S4; NT-proBNP: adjusted HR [95%CI], 1.749 [1.616–1.892], *P* < 0.001; creatinine: adjusted HR [95%CI], 1.744 [1.428–2.131], *P* < 0.001; albumin: adjusted HR [95%CI], 0.679 [0.570–0.809], *P* < 0.001). The multi-biomarker index, known as the CRH score, was developed based on the regression coefficients of these biomarkers (Additional file 1: Table S5), as below:

$$\mathrm{CRH}\;\mathrm{score}\hspace{0.17em}=\hspace{0.17em}0.669\hspace{0.17em}\times\hspace{0.17em}\ln\left(\mathrm{NT}-\mathrm{proBNP}\right)\hspace{0.17em}+\hspace{0.17em}0.245\hspace{0.17em}\times\hspace{0.17em}\ln\left(\mathrm{Creatinine}\right)\;-\;0.436\;\times\;\mathrm{Albumin}$$

where NT-proBNP was in pg/ml, creatinine in μmol/L, and albumin in g/dL. The median values of the CRH score were 3.86 (2.96–4.61), 3.47 (2.59–4.44), 3.55 (3.00–4.07), 4.22 (3.41–4.96), 4.20 (3.44–4.86), 4.64 (3.98–5.29), and 4.22 (3.41–4.96) in AS, AR, MS, MR, TR, MVHD, and total derivation cohort respectively, with detailed score distribution presented in Additional file 1: Figure S3.

The C index of the CRH score in total cohort was 0.78 (95%CI: 0.76–0.80), indicating high discrimination. The score also achieved satisfactory discrimination across all types of VHD (Additional file 1: Table S6; AS: 0.70 [0.61–0.80]; AR: 0.87 [0.84–0.91]; MS: 0.93 [0.88–0.97]; MR: 0.75 [0.71–0.79]; TR: 0.79 [0.75–0.83]; MVHD: 0.74 [0.71–0.77]). Regarding the calibration property of the CRH score, the calibration curves demonstrated excellent agreement between observed and predicted survival probabilities in both total cohort and various VHD (Additional file 1: Figure S4 and S5).

### Associated factors of cardio-renal-hepatic function

**Fig. 3:**
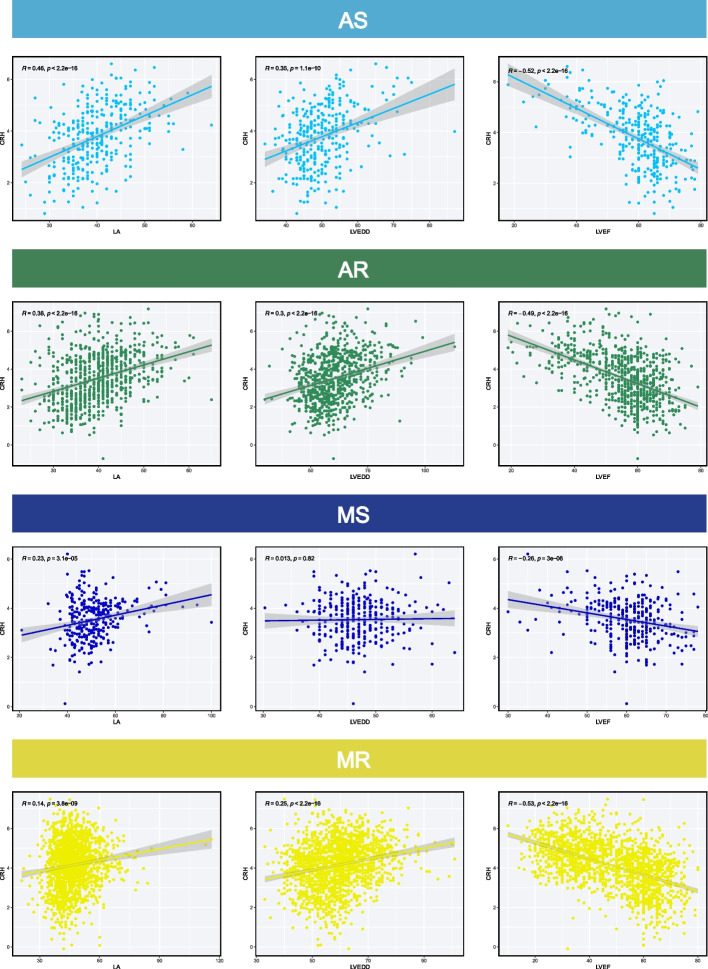
Relationship between CRH score and echocardiographic parameters in aortic and mitral valve diseases. The spearman correlations of CRH score with LA, LVEDD, and LVEF. AS, aortic stenosis; AR, aortic regurgitation; MS, mitral stenosis; MR, mitral regurgitation; LA, left atrial end-diastolic dimension; LVEDD, left ventricular end-diastolic dimension; LVEF, left ventricular ejection fraction; CRH, cardio-renal-hepatic

Correlations of left cardiac dimensions, function as well as other variables with the CRH score were presented in Fig. 3 and Additional file 1: Figure S6-S8. Significant positive correlations between left ventricular dimension and the score could be observed in AS, AR, MR, TR, and MVHD, while a negative correlation of LVEF with cardio-renal-hepatic function was found across all types of VHD (Fig. 3; Additional file 1: Figure S6). In multivariable analyses, LVEF was independently and negatively associated with the CRH score in all types of VHD (Additional file 1: Table S7), and was identified as the most important associated factor of cardio-renal-hepatic co-dysfunction in AS, AR, MR, TR, MVHD, as well as in total cohort (Additional file 1: Figure S9 and S10).

### Association of cardio-renal-hepatic function with mortality

**Fig. 4:**
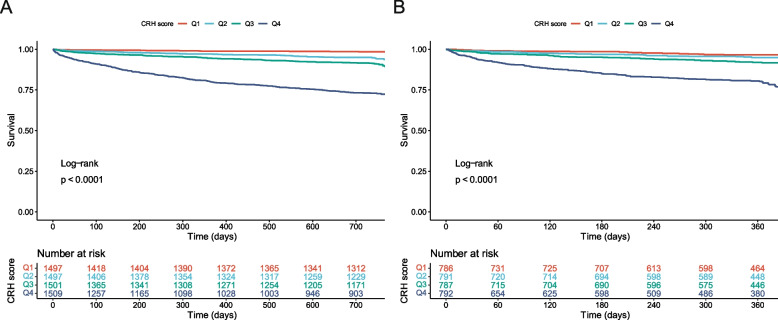
Kaplan–Meier curves according to quartiles of CRH score. **A** Kaplan–Meier curve in the derivation cohort. **B** Kaplan–Meier curve in the validation cohort. CRH, cardio-renal-hepatic

As a continuous variable, the CRH score was independently and strongly associated with mortality in overall population, with one-point increase carrying over two times of mortality risk (Table 3; overall adjusted HR [95% CI]: 2.095 [1.891–2.320], *P* < 0.001). The score was also a powerful predictor of mortality across all types of VHD in multivariable analyses (Table 3; adjusted HR [95%CI]: AS, 1.791 [1.018–3.148], *P* = 0.043; AR, 3.290 [2.245–4.821], *P* < 0.001; MS, 4.986 [2.069–12.016], *P* < 0.001; MR, 1.938 [1.573–2.389], *P* < 0.001; TR, 2.253 [1.808–2.808], *P* < 0.001; MVHD, 1.914 [1.603–2.284], *P* < 0.001). When analyzed categorically by quartile values, the score was significantly associated with mortality (Table 3; Fig. 4; Additional file 1: Figure S11; P_log-rank_ < 0.05 for all types of VHD).

**Table 3:** Associations of CRH score with mortality in patients with various VHD

	Univariable analysis		Multivariable analysis^a^	
	Unadjusted HR (95%CI)	*P* value	Adjusted HR (95%CI)	*P* value
**Total Cohort (** ***n*** ** = 6004)**				
CRH score (per 1 point increase)	2.719 (2.503–2.954)	< 0.001	2.095 (1.891–2.320)	< 0.001
CRH score				
Q2 vs Q1	3.636 (2.241–5.898)	< 0.001	2.586 (1.583–4.223)	< 0.001
Q3 vs Q1	6.293 (3.965–9.989)	< 0.001	3.652 (2.267–5.882)	< 0.001
Q4 vs Q1	21.246 (13.687–32.979)	< 0.001	8.319 (5.182–13.355)	< 0.001
**AS (** ***n*** ** = 328)**				
CRH score (per 1 point increase)	2.086 (1.448–3.004)	< 0.001	1.791 (1.018–3.148)	0.043
CRH score				
Q2 vs Q1	1.478 (0.247–8.848)	0.669	1.036 (0.157–6.813)	0.971
Q3 vs Q1	4.652 (1.005–21.538)	0.049	2.407 (0.473–12.240)	0.290
Q4 vs Q1	7.873 (1.792–34.584)	0.006	2.990 (0.546–16.380)	0.207
**AR (** ***n*** ** = 780)**				
CRH score (per 1 point increase)	3.416 (2.618–4.457)	< 0.001	3.290 (2.245–4.821)	< 0.001
CRH score				
Q2 vs Q1	—	—	—	—
Q3 vs Q1	—	—	—	—
Q4 vs Q1	—	—	—	—
**MS (** ***n*** ** = 320)** ^b^				
CRH score (per 1 point increase)	7.279 (3.457–15.320)	< 0.001	4.986 (2.069–12.016)	< 0.001
CRH score				
Q2 vs Q1	—	—	—	—
Q3 vs Q1	—	—	—	—
Q4 vs Q1	—	—	—	—
**MR (** ***n*** ** = 1677)**				
CRH score (per 1 point increase)	2.552 (2.161–3.014)	< 0.001	1.938 (1.573–2.389)	< 0.001
CRH score				
Q2 vs Q1	2.964 (1.260–6.973)	0.013	1.696 (0.708–4.064)	0.236
Q3 vs Q1	5.416 (2.414–12.148)	< 0.001	2.599 (1.118–6.044)	0.026
Q4 vs Q1	13.451 (6.216–29.106)	< 0.001	4.017 (1.720–9.381)	0.001
**TR (** ***n*** ** = 1085)**				
CRH score (per 1 point increase)	2.676 (2.246–3.188)	< 0.001	2.253 (1.808–2.808)	< 0.001
CRH score				
Q2 vs Q1	2.400 (0.922–6.247)	0.073	2.167 (0.816–5.752)	0.121
Q3 vs Q1	3.973 (1.611–9.798)	0.003	3.361 (1.318–8.571)	0.011
Q4 vs Q1	16.708 (7.293–38.279)	< 0.001	10.502 (4.315–25.557)	< 0.001
**MVHD (** ***n*** ** = 1814)**				
CRH score (per 1 point increase)	2.611 (2.255–3.024)	< 0.001	1.914 (1.603–2.284)	< 0.001
CRH score				
Q2 vs Q1	1.977 (1.104–3.539)	0.022	1.474 (0.817–2.657)	0.198
Q3 vs Q1	3.752 (2.190–6.429)	< 0.001	2.076 (1.182–3.647)	0.011
Q4 vs Q1	9.788 (5.904–16.228)	< 0.001	3.931 (2.258–6.844)	< 0.001

*CRH* cardio-renal-hepatic, *VHD* valvular heart disease, *AS* aortic stenosis, *AR* aortic regurgitation, *MS* mitral stenosis, *MR* mitral regurgitation, *TR* tricuspid regurgitation, *MVHD* multiple valvular heart disease, *BMI* body mass index, *NYHA* New York Heart Association, *LA* left atrial end-diastolic dimension, *LVEDD* left ventricular end-diastolic dimension, *LVEF* left ventricular ejection fraction, *HR* hazard ratio, *CI* confidence interval

^a^Adjusted for age, sex, BMI, smoking status, hypertension, hyperlipidemia, diabetes, prior myocardial infarction, cardiomyopathy, atrial fibrillation or flutter, chronic lung disease, NYHA functional class, hemoglobin, LA, LVEDD, LVEF, pulmonary hypertension, severity of VHD, and valvular intervention

^b^Adjusted for age and sex

### Association of cardio-renal-hepatic function with mortality in clinically meaningful subsets

During a median follow up of 730 (520.75–748) days, death occurred in 539 (13.2%) patients under medical treatment. The cumulative survival at one and two years were 90.1% and 85.6%, respectively. As in overall population, one-point increase of the CRH score was independently associated with more than two-fold risk of mortality in patients under conservative care (Table 4; adjusted HR [95%CI]: 2.168 [1.946–2.416], *P* < 0.001). The score was also an independent and powerful predictor of mortality in patients with AR (adjusted HR [95%CI]: 4.435 [2.781–7.072], *P* < 0.001), MS (adjusted HR [95%CI]: 4.401 [1.155–16.778], *P* = 0.030), MR (adjusted HR [95%CI]: 1.994 [1.601–2.483], *P* < 0.001), TR (adjusted HR [95%CI]: 2.200 [1.758–2.751], *P* < 0.001), and MVHD (adjusted HR [95%CI]: 1.977 [1.642–2.380], *P* < 0.001) under medical treatment. A borderline statistical significance was found in AS (adjusted HR [95%CI]: 2.499 [0.922–6.778], *P* = 0.072). The prognostic value of the CRH score was well retained in patients with primary VHD, as well as in most patients with LVEF ≥ 50% (AR, MS, MR, TR, and MVHD) (Table 4). In 1922 patients under valvular intervention, increasing level of the CRH score was also independently associated with higher risk of mortality (adjusted HR [95%CI]: 1.734 [1.220–2.464], *P* = 0.002). The survival benefit of valvular intervention over conservative therapy appeared to be constant across the range of the score (Additional file 1: Figure S12).

**Table 4:** Associations of CRH score with mortality in clinically meaningful subgroups of patients

	Multivariable analysis		
	Mortality under medical treatment	Mortality in patients with LVEF ≥ 50%	Mortality in patients with primary VHD
Total cohort			
Adjusted HR (95%CI)	2.168 (1.946–2.416)	2.072 (1.806–2.376)	2.199 (1.876–2.578)
*P* value	< 0.001	< 0.001	< 0.001
AS^a^			
Adjusted HR (95%CI)	2.499 (0.922–6.778)	1.387 (0.776–2.481)	1.791 (1.018–3.148)
*P* value	0.072	0.270	0.043
AR^b^			
Adjusted HR (95%CI)	4.435 (2.781–7.072)	3.615 (2.110–6.193)	3.289 (2.111–5.127)
*P* value	< 0.001	< 0.001	< 0.001
MS^c^			
Adjusted HR (95%CI)	4.401 (1.155–16.778)	10.749 (3.134–36.867)	4.986 (2.069–12.016)
*P* value	0.030	< 0.001	< 0.001
MR			
Adjusted HR (95%CI)	1.994 (1.601–2.483)	1.583 (1.112–2.253)	2.242 (1.445–3.477)
*P* value	< 0.001	0.011	< 0.001
TR			
Adjusted HR (95%CI)	2.200 (1.758–2.751)	2.581 (1.975–3.371)	3.602 (2.158–6.013)
*P* value	< 0.001	< 0.001	< 0.001
MVHD			
Adjusted HR (95%CI)	1.977 (1.642–2.380)	1.857 (1.449–2.378)	1.932 (1.485–2.514)
*P* value	< 0.001	< 0.001	< 0.001

Adjusted for age, sex, BMI, smoking status, hypertension, hyperlipidemia, diabetes, prior myocardial infarction, cardiomyopathy, atrial fibrillation or flutter, chronic lung disease, NYHA functional class, hemoglobin, LA, LVEDD, LVEF, pulmonary hypertension, severity of VHD, and valvular intervention

*CRH* cardio-renal-hepatic, *LVEF* left ventricular ejection fraction, *VHD* valvular heart disease, *AS* aortic stenosis, *AR* aortic regurgitation, *MS* mitral stenosis, *MR* mitral regurgitation, *TR* tricuspid regurgitation, *MVHD* multiple valvular heart disease, *BMI* body mass index, *NYHA* New York Heart Association, *LA* left atrial end-diastolic dimension, *LVEDD* left ventricular end-diastolic dimension, *HR* hazard ratio, *CI* confidence interval

^a^For AS patients under medical treatment, cardiomyopathy was not adjusted because no death occurred in patients with cardiomyopathy. For AS patients with LVEF ≥ 50%, cardiomyopathy and CLD were not adjusted because no death occurred in patients with cardiomyopathy or CLD

^b^For AR patients with LVEF ≥ 50%, cardiomyopathy was not adjusted because no death occurred in patients with cardiomyopathy

^c^Adjusted for age and sex

### Incremental prognostic information of the CRH score

The addition of the CRH score to the base predictive model substantially improved the prognostic capability of the model in the overall population (Additional file 1: Table S8; CRH score + base model vs base model, C index, 0.81 [0.80–0.83] vs 0.78 [0.76–0.80]; NRI [95%CI], 0.255 [0.204–0.299], *P *< 0.001; IDI [95%CI], 0.055 [0.038–0.073], *P* < 0.001; likelihood ratio test *P* < 0.001). The score also provided significantly incremental prognostic value over conventional clinical and echocardiographic variables, including left cardiac dimensions and function, in AS, AR, MR, TR, and MVHD (Additional file 1: Table S8; likelihood ratio test *P* < 0.05). A similar result was obtained in MS when added the score into the minimally adjusted model incorporating age and sex (Additional file 1: Table S8; likelihood ratio test *P* < 0.001). The decision curve analysis further demonstrated better clinical utility after the inclusion of the score to base models (Additional file 1: Figure S13 and S14). Notably, the cardio-renal-hepatic function, evaluating by the CRH score, showed significantly better predictive performance than the hepatorenal function, represented by the Model for End-Stage Liver Disease excluding international normalized ratio (MELD-XI) score (Additional file 1: Table S9). Additional analyses also confirmed that the novel score performed better than its components alone, especially for creatinine and albumin (Additional file 1: Table S10).

### Relative importance of cardio-renal-hepatic function

Relative importance of variables was evaluated by the proportion of explainable log-likelihood ratio χ^2^ statistics, best subset analysis, as well as the random survival forest. The cardio-renal-hepatic function, represented by the CRH score, was identified as the most important predictor of mortality in both total cohort and patients with AR, MS, MR, TR, and MVHD (Additional file 1: Figure S15-S19). In patients with AS, the importance of the score was ranked the second or third by different approaches, while previous myocardial infarction was consistently identified as the most contributive feature (Additional file 1: Figure S15-S19).

### External validation of the CRH score

During a median follow up of 364 (215–381) days, 272 (8.6%) deaths occurred in the China-DVD cohort. The cumulative survival at one year was 90.8%. The C index of the CRH score in the China-DVD cohort was 0.72 (95%CI: 0.69–0.75), indicating satisfactory discrimination. The score also exhibited adequate predictive performance in all types of VHD except MS (Additional file 1: Table S6; AS: 0.82 [0.73–0.91]; AR: 0.66 [0.52–0.81]; MS: 0.62 [0.37–0.87]; MR: 0.73 [0.67–0.78]; TR: 0.70 [0.61–0.79]; MVHD: 0.72 [0.67–0.76]). Calibration curves demonstrated excellent calibration of the score in both total cohort and various VHD (Additional file 1: Figure S4 and S20).

Per one-point increase of the CRH score, the relative risk of mortality increased by 85.0% in the China-DVD cohort (adjusted HR [95%CI]: 1.850 [1.592–2.151], *P* < 0.001). The score was also strongly associated with mortality in patients with AS (adjusted HR [95%CI], 2.633 [1.151–6.026], *P* = 0.022), AR (adjusted HR [95%CI], 2.004 [1.080–3.717], *P* = 0.028), MR (adjusted HR [95%CI], 1.656 [1.275–2.150], *P* < 0.001), TR (adjusted HR [95%CI], 2.169 [1.358–3.464], *P* = 0.001), and MVHD (adjusted HR [95%CI], 2.243 [1.721–2.924], *P* < 0.001), but not in those with MS (adjusted HR [95%CI], 0.625 [0.273–1.427], *P* = 0.264).

## Discussion

Using data from two large, contemporary, prospective cohorts, we developed and externally validated a multi-biomarker index, named the CRH score, to assess heart, kidney, and liver function in an integrative fashion, and analyzed the prognostic role of cardio-renal-hepatic function in patients with VHD. The CRH score achieved satisfactory discrimination and excellent calibration in two heterogeneous cohorts of VHD. The cardio-renal-hepatic function index correlated well with echocardiographic findings, and was an independent and powerful predictor of mortality. In most types of VHD, the cardio-renal-hepatic function substantially complemented traditional clinical and echocardiographic parameters in terms of predicting mortality risk, and was identified as the most important prognostic factor. The CRH score, which is calculated by three readily accessible biomarkers, provides a novel and pragmatic approach to assess cardio-renal-hepatic function in a prognostically meaningful manner, and may guide clinical management decisions in patients with VHD.

### Cardio-renal-hepatic interactions in VHD

A growing body of evidence implies the existence and development of multi-organ cross-talk in patents with VHD, most prominently the heart-liver and heart-kidney interactions [9, 11, 12, 14–20]. Results from early studies showed a significant association of elevated kidney or liver function biomarkers with the severity of VHD [11, 14, 31, 32], which might be attributed to increased systemic venous congestion or impaired cardiac output in patients with more severe valvular lesions and cardiac dysfunction. In recent years, studies focusing on patients under valvular intervention further demonstrated the direct contribution of VHD-induced damage to extra-cardiac organ, with data showing that the adverse remodeling and dysfunction of extra-cardiac organs could be reversible after valvular corrections [9, 12, 16, 18, 20, 33]. Although the mechanisms of “cardiorenal syndrome” and “cardiohepatic interaction” remain to be further elucidated in VHD, the kidney and liver function indexes have emerged as prognostic indicators [10, 12, 15–18, 28, 29]. However, given the relatively weak correlations of hepatorenal function indexes with echocardiographic findings in both prior analyses and the present study [17, 24], elevations of these parameters are unlikely to be mainly explained by the systemic consequences of cardiac dysfunction, or to reflect intrinsic changes of cardiac structure in patients with VHD. Therefore, measuring these indexes is insufficient to monitor progression of VHD, cardiac function, and systemic hemodynamic burden in reality. Expanding on previous findings, we proposed the concept of cardio-renal-hepatic co-dysfunction in patients with VHD, and hypothesized that it could promote better understanding of systemic hemodynamic impairments and improve risk stratification.

### Cardio-renal-hepatic co-dysfunction and CRH score

The VHD-related cardio-renal-hepatic co-dysfunction can be defined as a clinical syndrome. In this context, cardiac remodeling and dysfunction are induced or exacerbated by VHD, resulting in systemic venous congestion and decreased cardiac output, which further lead to the functional or structural impairments of liver and kidney. The most important feature of VHD-related cardio-renal-hepatic co-dysfunction is that if a successful valvular intervention is performed at an early stage, the structural and functional damages of heart, kidney, and liver will be reversible to some extent.

The multi-biomarker approach has been used to estimate event risk as well as identifying high-risk patients who tended to benefit from more intensive therapy in coronary artery disease [34, 35]. The merits of the multi-biomarker approach include its stable predictive value, user-friendly feature, as well as allowing an integrative consideration of multiple pathophysiological pathways of disease. To our best knowledge, the present study for the first time developed a novel multi-biomarker score with the integration of heart, kidney, and liver function biomarkers to enable assessment of multi-organ function, as well as the systemic condition in patients with VHD. The CRH score is a consequence-oriented multi-biomarker index which includes NT-proBNP, creatinine, and albumin as components quantifying cardio-renal-hepatic function. A prior study showed that NT-proBNP correlated well with echocardiographic parameters, and were independent prognostic factors in patients with significant AS, AR, MR, TR, and MVHD [24]. There was also evidence suggesting that renal and hepatic dysfunction, represented by the elevation of specific biomarkers such as creatinine, albumin, and bilirubin, played roles in prognostic evaluation in various VHD [8, 10, 15–18, 28, 29]. The hepatorenal function indexes, which combined renal and liver function biomarkers, were independently associated with outcomes in patients with AS, MR, TR, and MVHD [15–17, 28, 36, 37]. Currently, there is no approach measuring the cardio-renal-hepatic co-dysfunction, and no study evaluating its prognostic implications. Our study represents a novel step towards an ideal biomarker-based strategy for risk assessment in patients with VHD.

### Prognostic Importance of cardio-renal-hepatic function in VHD

The present study demonstrated that the CRH score was independently and strongly associated with all-cause mortality in patients with AS, AR, MR, TR, and MVHD, and provided substantially incremental prognostic information over traditional risk factors. In patients with MS, the predictive performance of CRH score was inconsistent between the derivation and validation cohorts, which could be attributed to the relatively small sample size and number of events. Given the significant prognostic value of the CRH score in the China-VHD cohort, we believe that the score is still a valuable index in MS, and should be further validated in larger cohorts.

One interesting finding of the present study was that the CRH score could predict mortality risk not only in the entire VHD population, but also in the subset with LVEF ≥ 50%. It is well established that left ventricular systolic dysfunction indicates poor outcome in patients with VHD [23]. However, patients with VHD and preserved LVEF can also be in different risk profiles, and there is evidence supporting the prognostic utility of blood parameters in these patients [17, 24–26]. Compared with LVEF, biomarker-based assessment may enable a more sensitive detection of early disease deterioration. In addition, although our results revealed the intimate relationship of LVEF with cardio-renal-hepatic function, as a cardiac index only assessing left ventricular systolic function, it is unlikely for LVEF to reflect systemic hemodynamic condition or prognosis better than the multi-biomarker index which allowed comprehensive evaluation of heart, kidney, and liver function.

In this study, we adopted both predictive modeling techniques and machine-learning approach to evaluate the relative importance of cardio-renal-hepatic function compared with other predictors. The CRH score was identified as the most powerful predictor in all types of VHD except AS, in which the score was also highly ranked as the second or third most important prognostic factor among clinical characteristics and echocardiographic findings. So far, there exists numerous studies investigating outcome determinants in patients with cardiovascular diseases. From the perspective of methodology, it is not difficult to identify new prognostic factors with independent prognostic value through traditional regression-based statistical analyses. However, the properties of an ideal marker are far beyond its independent prognostic effect among covariates, as the clinically useful marker should also be significantly more important and powerful than existing predictors, especially the determinants of current management decisions. There is scarce literature evaluating relative predictor importance of biomarkers in patients with VHD [17, 24]. Our previous analyses demonstrated that NT-proBNP was the most contributive prognostic factor among clinical characteristics and echocardiographic parameters in elderly patients with AS, AR, MR, and MVHD [24], and the hepatorenal function, measured by the modified MELD scores, was the most important predictor in patients with isolated TR [17]. The present study, using multiple methods including the machine-learning technique to evaluate variable importance, confirmed the crucial role of biomarker-based integrative cardio-renal-hepatic assessment in risk stratification in patients with VHD.

### Clinical implications of CRH score

The present study was far beyond proposing a novel concept, as it also provided important information to clinical management of VHD. Progressive multi-organ dysfunction is a crucial landmark of systemic hemodynamic deterioration in patients with VHD. It must be taken seriously because the adverse cardiac remodeling, symptoms of heart failure, as well as extra-cardiac organ impairments are not always reversible. In fact, once severe cardiac or hepatorenal failure occurs, patients are less likely to respond well to valvular corrections, regardless of operative approach [21, 22, 38]. This is particularly notable in patients with MR or TR [21–23, 36, 39, 40], and also merits attention in those with aortic valve disease and MVHD [8, 37, 41, 42]. The present study suggested that the CRH score could serve as a pragmatic tool to assess cardio-renal-hepatic function, and therefore could help identify high-risk patients as early as possible. Based on three readily accessible biomarkers, this multi-biomarker algorithm is easy to implement in routine clinical practice across all levels of medical institutions, which is of critical importance for monitoring disease progression closely [1].

### Limitations

The current study had several limitations. The addition of novel indexes to the present multi-biomarker score may provide new insights into cardio-renal-hepatic interactions and further improve the predictive performance of the score. Nevertheless, the CRH score has already included readily available biomarkers with ample evidence demonstrating their robust prognostic value in VHD. Serial measurements of cardio-renal-hepatic function may also further improve risk prediction in patients with VHD, which was not investigated in this study. However, the main purpose of this study was to develop a multi-biomarker index for assessing the cardio-renal-hepatic function as well as investigating its prognostic role. The association of longitudinal change in multi-organ function with outcomes will be an interesting topic for future investigations. In the multicenter cohort study, the NT-proBNP measurement was based on four assays, and the variation among laboratories was not evaluated. However, the four assays used same antibodies and calibrator from the same vendor, and previous data showed that the between-method variability of NT-proBNP was not the predominant component of total variability [43], and the imprecision performance of measurement of NT-proBNP in China had improved with the significant decrease of current coefficient of variations [44]. Finally, although the validation cohort of the present study allowed the CRH score to be externally tested in a population with a distinct age distribution, it had relatively shorter duration of follow up compared with the derivation cohort. More studies are needed to further validate this index.

## Conclusions

A novel multi-biomarker risk score was developed with three biomarkers evaluating heart, kidney, and liver function in patients with VHD. The CRH score, reflecting the systemic hemodynamic burden and multi-organ co-dysfunction in VHD, provided incremental prognostic information beyond clinical characteristics and echocardiographic findings. The score achieved satisfactory discrimination and calibration, and was validated in an external cohort. Biomarker-based assessment of cardio-renal-hepatic co-dysfunction is of particular importance to clinical risk stratification, and merits more attention in future research.

## Supplementary Information

**Additional file 1: Table S1.** Comparison of predictive performance among hepatic biomarkers. **Table S2.** Number of missing values and corresponding dispositions in the China-VHD cohort (*n* = 6004). **Table S3.** Number of missing values and corresponding dispositions in China-DVD cohort (*n* = 3156). **Table S4.** Associations of NT-proBNP, creatinine, and albumin with mortality in patients with various VHD. **Table S5.** Multivariable analysis of NT-proBNP, creatinine, and albumin. **Table S6.** Predictive performance of the CRH score in derivation and validation cohorts. **Table S7.** Associated factors of cardio-renal-hepatic co-dysfunction in China-VHD cohort. **Table S8.** Incremental value of CRH score beyond the base model. **Table S9.** Comparison of the CRH score with hepatorenal function index. **Table S10.** Comparison of CRH score with NT-proBNP, creatinine, and albumin. **Figure S1.** Flowchart of the derivation cohort. **Figure S2.** Flowchart of the validation cohort. **Figure S3.** The CRH score in different types of VHD. **Figure S4.** Calibration curves of CRH score in derivation and validation cohorts. **Figure S5.** Calibration curves of CRH score in different types of VHD in the derivation cohort. **Figure S6.** Relationship between CRH score and echocardiographic parameters in TR and MVHD in the derivation cohort. **Figure S7.** Correlation matrix in the derivation cohort. **Figure S8.** Correlation matrices in different types of VHD. **Figure S9.** Relative importance of predictors of cardio-renal-hepatic co-dysfunction in derivation and validation cohorts. **Figure S10.** Relative importance of predictors of cardio-renal-hepatic co-dysfunction in different types of VHD in derivation cohort. **Figure S11.** Kaplan–Meier curves according to types of VHD in the derivation cohort. **Figure S12.** Survival benefit of VI beyond MT according to CRH score. **Figure S13.** Decision curve analysis in the derivation cohort. **Figure S14.** Decision curve analysis in different types of VHD. **Figure S15.** Relative importance of predictors by the proportion of explainable log-likelihood ratio χ^2^ statistics in the derivation cohort. **Figure S16.** Relative importance of predictors by the proportion of explainable log-likelihood ratio χ^2^ statistics in different types of VHD. **Figure S17.** Relative importance of predictors by best subset analysis in the derivation cohort. **Figure S18.** Relative importance of predictors by best subset analysis in different types of VHD. **Figure S19.** Variable importance estimated by random survival forest. **Figure S20.** Calibration curves of CRH score in different types of VHD in the validation cohort.

**Additional file 2: Page S1.** Echocardiographic criteria of significant VHD in the China-VHD study. **Page S1.** Statistical analysis.

## Data Availability

The data used during the current study are available from the corresponding authors on reasonable request.

## Abbreviations

AR: Aortic regurgitation
AS: Aortic stenosis
CRH: Cardio-renal-hepatic
MR: Mitral regurgitation
MS: Mitral stenosis
MVHD: Multiple valvular heart disease
NT-proBNP: N-terminal pro-B-type natriuretic peptide
TR: Tricuspid regurgitation
VHD: Valvular heart disease
