# Supplementary material for: Assessment of cardio-renal-hepatic function in patients with valvular heart disease: a multi-biomarker approach—the cardio-renal-hepatic score
Source: BMC Med. 2023 Jul 17;21:257. doi: 10.1186/s12916-023-02971-y (PMC10351119; doi:10.1186/s12916-023-02971-y)
Supplement: Supplementary file 1 — Additional file 1: Table S1. Comparison of predictive performance among hepatic biomarkers. Table S2. Number of missing values and corresponding dispositions in the China-VHD cohort (n = 6004). Table S3. Number of missing values and corresponding dispositions in China-DVD cohort (n = 3156). Table S4. Associations of NT-proBNP, creatinine, and albumin with mortality in patients with various VHD. Table S5. Multivariable analysis of NT-proBNP, creatinine, and albumin. Table S6. Predictive performance of the CRH score in derivation and validation cohorts. Table S7. Associated factors of cardio-renal-hepatic co-dysfunction in China-VHD cohort. Table S8. Incremental value of CRH score beyond the base model. Table S9. Comparison of the CRH score with hepatorenal function index. Table S10. Comparison of CRH score with NT-proBNP, creatinine, and albumin. Figure S1. Flowchart of the derivation cohort. Figure S2. Flowchart of the validation cohort. Figure S3. The CRH score in different types of VHD. Figure S4. Calibration curves of CRH score in derivation and validation cohorts. Figure S5. Calibration curves of CRH score in different types of VHD in the derivation cohort. Figure S6. Relationship between CRH score and echocardiographic parameters in TR and MVHD in the derivation cohort. Figure S7. Correlation matrix in the derivation cohort. Figure S8. Correlation matrices in different types of VHD. Figure S9. Relative importance of predictors of cardio-renal-hepatic co-dysfunction in derivation and validation cohorts. Figure S10. Relative importance of predictors of cardio-renal-hepatic co-dysfunction in different types of VHD in derivation cohort. Figure S11. Kaplan–Meier curves according to types of VHD in the derivation cohort. Figure S12. Survival benefit of VI beyond MT according to CRH score. Figure S13. Decision curve analysis in the derivation cohort. Figure S14. Decision curve analysis in different types of VHD. Figure S15. Relative importance of predictors by the pro [file 12916_2023_2971_MOESM1_ESM.docx]

**Supplementary Tables.**

**Table S1. Comparison of predictive performance among hepatic biomarkers**

| Variables | C index (95%CI) | | | | | | |
| --- | --- | --- | --- | --- | --- | --- | --- |
|  | AS  (n=328) | AR  (n=780) | MS  (n=320) | MR  (n=1677) | TR  (n=1085) | MVHD  (n=1814) | Total cohort  (n=6004) |
| **Albumin** | **0.59 (0.49-0.70)** | **0.66 (0.57-0.74)** | 0.65 (0.45-0.86) | **0.62 (0.58-0.67)** | **0.68 (0.63-0.72)** | **0.64 (0.60-0.67)** | **0.65 (0.63-0.68)** |
| Total bilirubin | 0.50 (0.39-0.62) | 0.59 (0.50-0.68) | 0.75 (0.55-0.95) | 0.52 (0.47-0.57) | 0.51 (0.45-0.57) | 0.54 (0.50-0.58) | 0.54 (0.51-0.57) |
| Direct bilirubin | 0.51 (0.39-0.62) | 0.55 (0.46-0.64) | **0.81 (0.62-1.00)** | 0.55 (0.50-0.60) | 0.59 (0.54-0.64) | 0.61 (0.57-0.64) | 0.59 (0.57-0.62) |
| ALT | 0.54 (0.43-0.66) | 0.47 (0.37-0.58) | 0.51 (0.28-0.73) | 0.52 (0.46-0.57) | 0.46 (0.40-0.52) | 0.50 (0.46-0.54) | 0.50 (0.47-0.52) |

AS, aortic stenosis; AR, aortic regurgitation; MS, mitral stenosis; MR, mitral regurgitation; TR, tricuspid regurgitation; MVHD, multiple valvular heart disease; ALT, alanine aminotransferase; CI, confidence interval.

**Table S2. Number of missing values and corresponding dispositions in the China-VHD cohort (n=6004)**

|  | Number of missing values (%) | Disposition |
| --- | --- | --- |
| BMI | 184 (3.1) | Mean imputation |
| Hemoglobin | 30 (0.5) | Mean imputation |

VHD, valvular heart disease; BMI, body mass index.

**Table S3. Number of missing values and corresponding dispositions in China-DVD cohort (n=3156)**

|  | Number of missing values (%) | Disposition |
| --- | --- | --- |
| NYHA functional class | 18 (0.6) | Excluded in multivariable analysis |
| Hemoglobin | 15 (0.5) | Mean imputation |
| LA | 87 (2.8) | Mean imputation |
| LVEDD | 110 (3.5) | Mean imputation |
| LVEF | 1 (0.03) | Median imputation |

China-DVD, China Elderly Valve Disease; NYHA, New York Heart Association; LA, left atrial end-diastolic dimension; LVEDD, left ventricular end-diastolic dimension; LVEF, left ventricular ejection fraction.

**Table S4. Associations of NT-proBNP, creatinine, and albumin with mortality in patients with various VHD**

|  | Univariable analysis | |  | Multivariable analysis^*^ | |
| --- | --- | --- | --- | --- | --- |
|  | Unadjusted HR (95%CI) | P value |  | Adjusted HR (95%CI) | P value |
| **Total cohort (n=6004)** | | | | | |
| ln(NT-proBNP) (per 1 increase) | 2.122 (1.987-2.265) | <0.001 |  | 1.749 (1.616-1.892) | <0.001 |
| ln(Creatinine) (per 1 increase) | 3.210 (2.724-3.783) | <0.001 |  | 1.744 (1.428-2.131) | <0.001 |
| Albumin (per 1g/dl increase) | 0.393 (0.343-0.451) | <0.001 |  | 0.679 (0.570-0.809) | <0.001 |
| **AS (n=328)** | | | | | |
| ln(NT-proBNP) (per 1 increase) | 1.632 (1.260-2.115) | <0.001 |  | 1.442 (0.982-2.117) | 0.062 |
| ln(Creatinine) (per 1 increase) | 3.021 (0.928-9.836) | 0.066 |  | 2.988 (0.735-12.140) | 0.126 |
| Albumin (per 1g/dl increase) | 0.476 (0.234-0.969) | 0.041 |  | 0.800 (0.360-1.777) | 0.584 |
| **AR (n=780)** | | | | | |
| ln(NT-proBNP) (per 1 increase) | 2.437 (1.994-2.978) | <0.001 |  | 2.411 (1.825-3.185) | <0.001 |
| ln(Creatinine) (per 1 increase) | 5.674 (2.805-11.477) | <0.001 |  | 1.666 (0.652-4.257) | 0.286 |
| Albumin (per 1g/dl increase) | 0.209 (0.110-0.399) | <0.001 |  | 0.565 (0.241-1.325) | 0.189 |
| **MS (n=320)**^†^ | | | | | |
| ln(NT-proBNP) (per 1 increase) | 4.134 (2.375-7.196) | <0.001 |  | 3.024 (1.629-5.613) | <0.001 |
| ln(Creatinine) (per 1 increase) | 13.066 (1.250-136.544) | 0.032 |  | 8.895 (0.0.441-179.615) | 0.154 |
| Albumin (per 1g/dl increase) | 0.226 (0.057-0.900) | 0.035 |  | 0.457 (0.104-2.004) | 0.299 |
| **MR (n=1677)** | | | | | |
| ln(NT-proBNP) (per 1 increase) | 2.061 (1.806-2.352) | <0.001 |  | 1.677 (1.424-1.976) | <0.001 |
| ln(Creatinine) (per 1 increase) | 2.647 (1.899-3.690) | <0.001 |  | 1.773 (1.167-2.694) | 0.007 |
| Albumin (per 1g/dl increase) | 0.502 (0.386-0.654) | <0.001 |  | 0.783 (0.565-1.086) | 0.143 |
| **TR (n=1085)** | | | | | |
| ln(NT-proBNP) (per 1 increase) | 2.109 (1.829-2.432) | <0.001 |  | 1.889 (1.583-2.254) | <0.001 |
| ln(Creatinine) (per 1 increase) | 3.044 (2.203-4.204) | <0.001 |  | 1.793 (1.196-2.690) | 0.005 |
| Albumin (per 1g/dl increase) | 0.340 (0.251-0.461) | <0.001 |  | 0.580 (0.397-0.847) | 0.005 |
| **MVHD (n=1814)** | | | | | |
| ln(NT-proBNP) (per 1 increase) | 2.051 (1.825-2.304) | <0.001 |  | 1.602 (1.401-1.833) | <0.001 |
| ln(Creatinine) (per 1 increase) | 3.141 (2.400-4.110) | <0.001 |  | 1.674 (1.217-2.303) | 0.002 |
| Albumin (per 1g/dl increase) | 0.427 (0.331-0.552) | <0.001 |  | 0.730 (0.543-0.981) | 0.037 |

^*^Adjusted for age, sex, BMI, smoking status, hypertension, hyperlipidemia, diabetes, previous myocardial infarction, cardiomyopathy, atrial fibrillation or flutter, chronic lung disease, NYHA functional class, hemoglobin, LA, LVEDD, LVEF, pulmonary hypertension, severity of VHD, and valvular intervention. ^†^Adjusted for age and sex. NT-proBNP, N-terminal pro-B-type natriuretic peptide; VHD, valvular heart disease; AS, aortic stenosis; AR, aortic regurgitation; MS, mitral stenosis; MR, mitral regurgitation; TR, tricuspid regurgitation; MVHD, multiple valvular heart disease; BMI, body mass index; NYHA, New York Heart Association; LA, left atrial end-diastolic dimension; LVEDD, left ventricular end-diastolic dimension; LVEF, left ventricular ejection fraction; HR, hazard ratio; CI, confidence interval.

**Table S5. Multivariable analysis of NT-proBNP, creatinine, and albumin**

|  | Coefficient | HR (95%CI) | P value |
| --- | --- | --- | --- |
| ln(NT-proBNP) (per 1 increase) | 0.669 | 1.952 (1.815-2.100) | <0.001 |
| ln(Creatinine) (per 1 increase) | 0.245 | 1.277 (1.057-1.544) | 0.011 |
| Albumin (per 1g/dl increase) | -0.436 | 0.646 (0.549-0.761) | <0.001 |

NT-proBNP, N-terminal pro-B-type natriuretic peptide; HR, hazard ratio; CI, confidence interval.

**Table S6. Predictive performance of the CRH score in two cohorts**

| Types of VHD | C index (95%CI) | |
| --- | --- | --- |
|  | China-VHD | China-DVD |
| Total cohort | 0.78 (0.76-0.80) | 0.72 (0.69-0.75) |
| AS | 0.70 (0.61-0.80) | 0.82 (0.73-0.91) |
| AR | 0.87 (0.84-0.91) | 0.66 (0.52-0.81) |
| MS | 0.93 (0.88-0.97) | 0.62 (0.37-0.87) |
| MR | 0.75 (0.71-0.79) | 0.73 (0.67-0.78) |
| TR | 0.79 (0.75-0.83) | 0.70 (0.61-0.79) |
| MVHD | 0.74 (0.71-0.77) | 0.72 (0.67-0.76) |

CRH, cardio-renal-hepatic; VHD, valvular heart disease; China-DVD, China Elderly Valve Disease; AS, aortic stenosis; AR, aortic regurgitation; MS, mitral stenosis; MR, mitral regurgitation; TR, tricuspid regurgitation; MVHD, multiple valvular heart disease; CI, confidence interval.

**Table S7. Associated factors of cardio-renal-hepatic co-dysfunction in China-VHD cohort**

| Variables | Coefficient | SE | Standardized Coefficient | P Value |
| --- | --- | --- | --- | --- |
| **Total cohort (n=6004)** | | | | |
| **Age (per 1 year increase)** | **0.0129910** | **0.0009636** | **0.1523326035** | **<0.001** |
| **Female (vs male)** | **0.0730331** | **0.0273995** | **0.0307472549** | **0.008** |
| **BMI (per 1kg/m^2^ increase)** | **-0.0262391** | **0.0033555** | **-0.0809862294** | **<0.001** |
| Current smoker (vs no) | 0.0316869 | 0.0334127 | 0.0099744970 | 0.343 |
| **Hypertension (vs no)** | **0.0719992** | **0.0257540** | **0.0303973565** | **0.005** |
| **Hyperlipidemia (vs no)** | **-0.1059803** | **0.0345932** | **-0.0310141224** | **0.002** |
| Diabetes (vs no) | -0.0004601 | 0.0338777 | -0.0001413084 | 0.989 |
| **Myocardial infarction (vs no)** | **0.1774976** | **0.0391092** | **0.0477828233** | **<0.001** |
| **Cardiomyopathy (vs no)** | **0.1300510** | **0.0417323** | **0.0336632760** | **0.002** |
| **Atrial fibrillation or flutter (vs no)** | **0.1732759** | **0.0284676** | **0.0674823696** | **<0.001** |
| **Chronic lung disease (vs no)** | **0.1201792** | **0.0463270** | **0.0262559667** | **0.010** |
| **NYHA III-IV (vs I-II)** | **0.3775729** | **0.0250870** | **0.1603202937** | **<0.001** |
| **Hemoglobin (per 1g/L increase)** | **-0.0092888** | **0.0006155** | **-0.1636653497** | **<0.001** |
| **LA (per 1mm increase)** | **0.0077255** | **0.0014302** | **0.0637689105** | **<0.001** |
| **LVEDD (per 1mm increase)** | **-0.0032086** | **0.0014110** | **-0.0309464791** | **0.023** |
| **LVEF (per 1% increase)** | **-0.0341832** | **0.0011148** | **-0.4024601019** | **<0.001** |
| **Pulmonary hypertension (vs no)** | **0.3823254** | **0.0248322** | **0.1607358224** | **<0.001** |
| **Severe VHD (vs moderate VHD)** | **0.1442594** | **0.0248582** | **0.0609854983** | **<0.001** |
| **AS (n=328)** | | | | |
| **Age (per 1 year increase)** | **0.015335** | **0.004904** | **0.15096619** | **0.002** |
| Female (vs male) | 0.045565 | 0.113853 | 0.01946354 | 0.689 |
| **BMI (per 1kg/m^2^ increase)** | **-0.045634** | **0.015698** | **-0.13117317** | **0.004** |
| Current smoker (vs no) | -0.193499 | 0.129042 | -0.06599254 | 0.135 |
| Hypertension (vs no) | -0.085965 | 0.108909 | -0.03696717 | 0.431 |
| Hyperlipidemia (vs no) | -0.048423 | 0.116581 | -0.01803627 | 0.678 |
| Diabetes (vs no) | 0.044919 | 0.143987 | 0.01346209 | 0.755 |
| Myocardial infarction (vs no) | 0.129935 | 0.216731 | 0.02577639 | 0.549 |
| Cardiomyopathy (vs no) | 0.607871 | 0.441256 | 0.05811568 | 0.169 |
| Atrial fibrillation or flutter (vs no) | 0.225169 | 0.199236 | 0.04801378 | 0.259 |
| Chronic lung disease (vs no) | 0.225002 | 0.233596 | 0.04221857 | 0.336 |
| NYHA III-IV (vs I-II) | 0.168633 | 0.097929 | 0.07342379 | 0.086 |
| **Hemoglobin (per 1g/L increase)** | **-0.009166** | **0.002986** | **-0.14405277** | **0.002** |
| **LA (per 1mm increase)** | **0.041628** | **0.009082** | **0.22956770** | **<0.001** |
| **LVEDD (per 1mm increase)** | **0.022607** | **0.008226** | **0.14476567** | **0.006** |
| **LVEF (per 1% increase)** | **-0.033475** | **0.005606** | **-0.30401651** | **<0.001** |
| Pulmonary hypertension (vs no) | 0.296794 | 0.156307 | 0.08274170 | 0.059 |
| **Severe AS (vs moderate AS)** | **0.531752** | **0.118528** | **0.19543166** | **<0.001** |
| **AR (n=780)** | | | | |
| **Age (per 1 year increase)** | **0.018898** | **0.002838** | **0.211267120** | **<0.001** |
| Female (vs male) | 0.068825 | 0.092973 | 0.022520232 | 0.459 |
| **BMI (per 1kg/m^2^ increase)** | **-0.043475** | **0.010745** | **-0.114644594** | **<0.001** |
| Current smoker (vs no) | 0.020750 | 0.083403 | 0.006775732 | 0.804 |
| **Hypertension (vs no)** | **0.149489** | **0.071388** | **0.058223078** | **0.037** |
| Hyperlipidemia (vs no) | -0.028955 | 0.087969 | -0.008805950 | 0.742 |
| Diabetes (vs no) | -0.119990 | 0.115338 | -0.028131059 | 0.299 |
| Myocardial infarction (vs no) | 0.224520 | 0.127160 | 0.048430502 | 0.078 |
| Cardiomyopathy (vs no) | 0.091697 | 0.183409 | 0.013407841 | 0.617 |
| **Atrial fibrillation or flutter (vs no)** | **0.479538** | **0.120883** | **0.109099663** | **<0.001** |
| **Chronic lung disease (vs no)** | **0.438399** | **0.135275** | **0.087468755** | **0.001** |
| **NYHA III-IV (vs I-II)** | **0.242311** | **0.079141** | **0.085937785** | **0.002** |
| **Hemoglobin (per 1g/L increase)** | **-0.015393** | 0.002022 | **-0.225806596** | **<0.001** |
| **LA (per 1mm increase)** | **0.036642** | **0.006245** | **0.184885584** | **<0.001** |
| **LVEDD (per 1mm increase)** | **0.019836** | **0.004614** | **0.161254148** | **<0.001** |
| **LVEF (per 1% increase)** | **-0.033805** | **0.004098** | **-0.270295770** | **<0.001** |
| **Pulmonary hypertension (vs no)** | **0.497246** | **0.125489** | **0.106492490** | **<0.001** |
| **Severe AR (vs moderate AR)** | **0.257937** | **0.079091** | **0.097908772** | **0.001** |
| **MS (n=320)** | | | | |
| **Age (per 1 year increase)** | **0.020213** | **0.004107** | **0.27371912** | **<0.001** |
| Female (vs male) | 0.068073 | 0.103560 | 0.03815074 | 0.511 |
| **BMI (per 1kg/m^2^ increase)** | **-0.026423** | **0.012704** | **-0.10555437** | **0.038** |
| Current smoker (vs no) | 0.063089 | 0.158360 | 0.02108080 | 0.691 |
| Hypertension (vs no) | -0.115911 | 0.109464 | -0.05636789 | 0.290 |
| Hyperlipidemia (vs no) | -0.054964 | 0.113376 | -0.02358321 | 0.628 |
| Diabetes (vs no) | -0.014477 | 0.158500 | -0.00457289 | 0.927 |
| Myocardial infarction (vs no) | 0.419644 | 0.301122 | 0.06961340 | 0.164 |
| **Atrial fibrillation or flutter (vs no)** | **0.426106** | **0.087334** | **0.25941276** | **<0.001** |
| Chronic lung disease (vs no) | 0.551152 | 0.294056 | 0.09142869 | 0.062 |
| NYHA III-IV (vs I-II) | 0.140643 | 0.079278 | 0.08485879 | 0.077 |
| **Hemoglobin (per 1g/L increase)** | **-0.008246** | **0.002689** | **-0.16525210** | **0.002** |
| **LA (per 1mm increase)** | **0.009719** | **0.004590** | **0.11336166** | **0.035** |
| LVEDD (per 1mm increase) | -0.008730 | 0.008307 | -0.05380217 | 0.294 |
| **LVEF (per 1% increase)** | **-0.018182** | **0.005149** | **-0.17669229** | **<0.001** |
| **Pulmonary hypertension (vs no)** | **0.203785** | **0.081759** | **0.12193383** | **0.013** |
| Severe MS (vs moderate MS) | 0.104326 | 0.084148 | 0.06334303 | 0.216 |
| **MR (n=1677)** | | | | |
| **Age (per 1 year increase)** | **0.013964** | **0.001949** | **0.148325307** | **<0.001** |
| Female (vs male) | 0.070661 | 0.050922 | 0.028908004 | 0.165 |
| **BMI (per 1kg/m^2^ increase)** | **-0.025333** | **0.006308** | **-0.076106963** | **<0.001** |
| **Current smoker (vs no)** | **0.112150** | **0.056045** | **0.038921036** | **0.046** |
| Hypertension (vs no) | 0.076363 | 0.046320 | 0.032032632 | 0.099 |
| **Hyperlipidemia (vs no)** | **-0.139886** | **0.060523** | **-0.043389603** | **0.021** |
| Diabetes (vs no) | 0.070118 | 0.057851 | 0.023340906 | 0.226 |
| **Myocardial infarction (vs no)** | **0.140574** | **0.059617** | **0.046018070** | **0.018** |
| **Cardiomyopathy (vs no)** | **0.247446** | **0.062322** | **0.078302208** | **<0.001** |
| **Atrial fibrillation or flutter (vs no)** | **0.208410** | **0.057991** | **0.072399007** | **<0.001** |
| Chronic lung disease (vs no) | -0.040179 | 0.096651 | -0.007726073 | 0.678 |
| **NYHA III-IV (vs I-II)** | **0.346409** | **0.047171** | **0.145493152** | **<0.001** |
| **Hemoglobin (per 1g/L increase)** | **-0.013116** | **0.001227** | **-0.220672167** | **<0.001** |
| **LA (per 1mm increase)** | **0.022979** | **0.003115** | **0.165011578** | **<0.001** |
| **LVEDD (per 1mm increase)** | **-0.013542** | **0.003115** | **-0.114427424** | **<0.001** |
| **LVEF (per 1% increase)** | **-0.036614** | **0.002031** | **-0.464214247** | **<0.001** |
| **Pulmonary hypertension (vs no)** | **0.318482** | **0.049019** | **0.121834826** | **<0.001** |
| Severe MR (vs moderate MR) | -0.094298 | 0.050852 | -0.036741201 | 0.064 |
| **TR (n=1085)** | | | | |
| **Age (per 1 year increase)** | **0.006366** | **0.002096** | **0.096202308** | **0.002** |
| Female (vs male) | 0.030691 | 0.065044 | 0.013782843 | 0.637 |
| **BMI (per 1kg/m^2^ increase)** | **-0.014982** | **0.007302** | **-0.054654701** | **0.040** |
| Current smoker (vs no) | -0.012193 | 0.095050 | -0.003463972 | 0.898 |
| Hypertension (vs no) | 0.126672 | 0.064786 | 0.056344367 | 0.051 |
| **Hyperlipidemia (vs no)** | **-0.256897** | **0.095832** | **-0.068881969** | **0.007** |
| Diabetes (vs no) | 0.018694 | 0.081382 | 0.006051848 | 0.818 |
| **Myocardial infarction (vs no)** | **0.357280** | **0.104593** | **0.092101222** | **<0.001** |
| Cardiomyopathy (vs no) | 0.193138 | 0.119960 | 0.045129835 | 0.108 |
| Atrial fibrillation or flutter (vs no) | 0.043198 | 0.066028 | 0.018817766 | 0.513 |
| Chronic lung disease (vs no) | 0.146028 | 0.091112 | 0.042647455 | 0.109 |
| **NYHA III-IV (vs I-II)** | **0.516041** | **0.061519** | **0.228026706** | **<0.001** |
| **Hemoglobin (per 1g/L increase)** | **-0.005615** | **0.001245** | **-0.125801815** | **<0.001** |
| **LA (per 1mm increase)** | **0.009201** | **0.004222** | **0.069857290** | **0.030** |
| **LVEDD (per 1mm increase)** | **-0.014224** | **0.004527** | **-0.113605306** | **0.002** |
| **LVEF (per 1% increase)** | **-0.031783** | **0.002990** | **-0.344405952** | **<0.001** |
| **Pulmonary hypertension (vs no)** | **0.325419** | **0.060263** | **0.142031318** | **<0.001** |
| **Severe TR (vs moderate TR)** | **0.226904** | **0.063468** | **0.095174382** | **<0.001** |
| **MVHD (n=1814)** | | | | |
| **Age (per 1 year increase)** | **0.011953** | **0.001659** | **0.154898396** | **<0.001** |
| **Female (vs male)** | **0.142054** | **0.044825** | **0.070529767** | **0.002** |
| **BMI (per 1kg/m^2^ increase)** | **-0.022908** | **0.005687** | **-0.081509013** | **<0.001** |
| Current smoker (vs no) | 0.079664 | 0.060486 | 0.026678736 | 0.188 |
| Hypertension (vs no) | 0.035488 | 0.043691 | 0.017423979 | 0.417 |
| Hyperlipidemia (vs no) | 0.029570 | 0.066081 | 0.008734921 | 0.655 |
| Diabetes (vs no) | -0.047067 | 0.055770 | -0.017147335 | 0.399 |
| Myocardial infarction (vs no) | 0.134257 | 0.068498 | 0.039957118 | 0.050 |
| Cardiomyopathy (vs no) | 0.021390 | 0.065258 | 0.006991892 | 0.743 |
| **Atrial fibrillation or flutter (vs no)** | **0.139871** | **0.042682** | **0.069007135** | **0.001** |
| Chronic lung disease (vs no) | -0.009519 | 0.076311 | -0.002426836 | 0.901 |
| **NYHA III-IV (vs I-II)** | **0.354276** | **0.041427** | **0.172589467** | **<0.001** |
| **Hemoglobin (per 1g/L increase)** | **-0.009719** | **0.001027** | **-0.195810347** | **<0.001** |
| **LA (per 1mm increase)** | **-0.005874** | **0.002233** | **-0.057563499** | **0.009** |
| **LVEDD (per 1mm increase)** | **0.006764** | **0.002319** | **0.076099874** | **0.004** |
| **LVEF (per 1% increase)** | **-0.026566** | **0.001792** | **-0.374084207** | **<0.001** |
| **Pulmonary hypertension (vs no)** | **0.205091** | **0.041172** | **0.097730441** | **<0.001** |
| **Severe MVHD (vs moderate MVHD)** | **0.107647** | **0.041608** | **0.052340279** | **0.010** |

China-VHD, China Valvular Heart Disease; BMI, body mass index; NYHA, New York Heart Association; LA, left atrial end-diastolic dimension; LVEDD, left ventricular end-diastolic dimension; LVEF, left ventricular ejection fraction; VHD, valvular heart disease; AS, aortic stenosis; AR, aortic regurgitation; MS, mitral stenosis; MR, mitral regurgitation; TR, tricuspid regurgitation; MVHD, multiple valvular heart disease; SE, standard error.

**Table S8. Incremental value of CRH score beyond the base model**

|  | Base model^*^ | Base model+CRH score |
| --- | --- | --- |
| **Total cohort (n=6004)** | | |
| C index | 0.78 (0.76-0.80) | 0.81 (0.80-0.83) |
| NRI (95%CI) | 0.255 (0.204-0.299) | |
| P value | <0.001 | |
| IDI (95%CI) | 0.055 (0.038-0.073) | |
| P value | <0.001 | |
| Likelihood ratio test (P value) | <0.001 | |
| BIC | 9564.10 | 9362.41 |
| **AS (n=328)** | | |
| C index | 0.81 (0.73-0.89) | 0.82 (0.75-0.90) |
| NRI (95%CI) | 0.159 (-0.166-0.403) | |
| P value | 0.254 | |
| IDI (95%CI) | 0.017 (-0.008-0.077) | |
| P value | 0.158 | |
| Likelihood ratio test (P value) | 0.035 | |
| BIC | 342.47 | 341.39 |
| **AR (n=780)** | | |
| C index | 0.86 (0.81-0.91) | 0.90 (0.86-0.94) |
| NRI (95%CI) | 0.414 (0.187-0.561) | |
| P value | <0.001 | |
| IDI (95%CI) | 0.151 (0.060-0.231) | |
| P value | <0.001 | |
| Likelihood ratio test (P value) | <0.001 | |
| BIC | 526.04 | 487.80 |
| **MS (n=320)** | | |
| C index | 0.79 (0.65-0.94) | 0.94 (0.90-0.98) |
| NRI (95%CI) | 0.332 (-0.206-0.746) | |
| P value | 0.098 | |
| IDI (95%CI) | 0.113 (-0.050-0.208) | |
| P value | 0.082 | |
| Likelihood ratio test (P value) | <0.001 | |
| BIC | 93.17 | 81.32 |
| **MR (n=1677)** | | |
| C index | 0.77 (0.74-0.81) | 0.80 (0.76-0.83) |
| NRI (95%CI) | 0.203 (0.104-0.293) | |
| P value | <0.001 | |
| IDI (95%CI) | 0.041 (0.018-0.074) | |
| P value | <0.001 | |
| Likelihood ratio test (P value) | <0.001 | |
| BIC | 2061.52 | 2026.77 |
| **TR (n=1085)** | | |
| C index | 0.78 (0.74-0.82) | 0.82 (0.79-0.86) |
| NRI (95%CI) | 0.319 (0.201-0.432) | |
| P value | <0.001 | |
| IDI (95%CI) | 0.076 (0.039-0.124) | |
| P value | <0.001 | |
| Likelihood ratio test (P value) | <0.001 | |
| BIC | 1626.18 | 1574.69 |
| **MVHD (n=1814)** | | |
| C index | 0.78 (0.75-0.81) | 0.80 (0.77-0.83) |
| NRI (95%CI) | 0.210 (0.119-0.290) | |
| P value | <0.001 | |
| IDI (95%CI) | 0.044 (0.021-0.074) | |
| P value | <0.001 | |
| Likelihood ratio test (P value) | <0.001 | |
| BIC | 3391.15 | 3343.17 |

^*^Base model for AS, AR, MR, TR, and MVHD: age, sex, BMI, smoking status, hypertension, hyperlipidemia, diabetes, previous myocardial infarction, cardiomyopathy, atrial fibrillation or flutter, chronic lung disease, NYHA functional class, hemoglobin, LA, LVEDD, LVEF, pulmonary hypertension, severity of VHD, and valvular intervention. Base model for MS: age and sex. CRH, cardio-renal-hepatic; NRI, net reclassification index; IDI, integrated discrimination improvement index; BIC, Bayesian information criterion; AS, aortic stenosis; AR, aortic regurgitation; MS, mitral stenosis; MR, mitral regurgitation; TR, tricuspid regurgitation; MVHD, multiple valvular heart disease; BMI, body mass index; NYHA, New York Heart Association; LA, left atrial end-diastolic dimension; LVEDD, left ventricular end-diastolic dimension; LVEF, left ventricular ejection fraction; VHD, valvular heart disease; CI, confidence interval.

**Table S9. Comparison of the CRH score with hepatorenal function index**

|  | MELD-XI score | CRH score |
| --- | --- | --- |
| **Total cohort (n=6004)** | | |
| C index | 0.65 (0.62-0.67) | 0.78 (0.76-0.80) |
| NRI (95%CI) | 0.364 (0.303-0.411) | |
| P value | <0.001 | |
| IDI (95%CI) | 0.094 (0.074-0.115) | |
| P value | <0.001 | |
| BIC | 9935.96 | 9476.02 |
| **AS (n=328)** | | |
| C index | 0.52 (0.42-0.62) | 0.70 (0.61-0.80) |
| NRI (95%CI) | 0.269 (0.081-0.429) | |
| P value | 0.010 | |
| IDI (95%CI) | 0.057 (0.009-0.142) | |
| P value | 0.020 | |
| BIC | 323.86 | 308.49 |
| **AR (n=780)** | | |
| C index | 0.67 (0.58-0.75) | 0.87 (0.84-0.91) |
| NRI (95%CI) | 0.661 (0.488-0.729) | |
| P value | <0.001 | |
| IDI (95%CI) | 0.171 (0.096-0.261) | |
| P value | <0.001 | |
| BIC | 534.50 | 457.57 |
| **MS (n=320)** | | |
| C index | 0.83 (0.68-0.98) | 0.93 (0.88-0.97) |
| NRI (95%CI) | 0.363 (-0.105-0.723) | |
| P value | 0.138 | |
| IDI (95%CI) | 0.123 (-0.007-0.242) | |
| P value | 0.064 | |
| BIC | 92.43 | 79.02 |
| **MR (n=1677)** | | |
| C index | 0.62 (0.57-0.66) | 0.75 (0.71-0.79) |
| NRI (95%CI) | 0.323 (0.197-0.422) | |
| P value | <0.001 | |
| IDI (95%CI) | 0.084 (0.049-0.126) | |
| P value | <0.001 | |
| BIC | 2103.73 | 1992.33 |
| **TR (n=1085)** | | |
| C index | 0.65 (0.60-0.70) | 0.79 (0.75-0.83) |
| NRI (95%CI) | 0.437 (0.339-0.548) | |
| P value | <0.001 | |
| IDI (95%CI) | 0.099 (0.061-0.145) | |
| P value | <0.001 | |
| BIC | 1648.84 | 1555.59 |
| **MVHD (n=1814)** | | |
| C index | 0.64 (0.60-0.68) | 0.74 (0.71-0.77) |
| NRI (95%CI) | 0.293 (0.172-0.388) | |
| P value | <0.001 | |
| IDI (95%CI) | 0.074 (0.042-0.112) | |
| P value | <0.001 | |
| BIC | 3481.52 | 3367.31 |

CRH, cardio-renal-hepatic; MELD-XI, Model for End-Stage Liver Disease excluding international normalized ratio; NRI, net reclassification index; IDI, integrated discrimination improvement index; BIC, Bayesian information criterion; AS, aortic stenosis; AR, aortic regurgitation; MS, mitral stenosis; MR, mitral regurgitation; TR, tricuspid regurgitation; MVHD, multiple valvular heart disease; CI, confidence interval.

**Table S10. Comparison of CRH score** with NT-proBNP, creatinine, and albumin

|  | ln(NT-proBNP) | CRH score |
| --- | --- | --- |
| C index | 0.77 (0.75-0.79) | 0.78 (0.76-0.80) |
| NRI (95%CI) | 0.082 (-0.009-0.169) | |
| P value | 0.088 | |
| IDI (95%CI) | 0.008 (0.001-0.016) | |
| P value | 0.030 | |
| BIC | 9512.06 | 9476.02 |
|  | ln(Creatinine) | CRH score |
| C index | 0.62 (0.60-0.65) | 0.78 (0.76-0.80) |
| NRI (95%CI) | 0.380 (0.327-0.425) | |
| P value | <0.001 | |
| IDI (95%CI) | 0.097 (0.077-0.118) | |
| P value | <0.001 | |
| BIC | 9958.04 | 9476.02 |
|  | Albumin | CRH score |
| C index | 0.65 (0.63-0.68) | 0.78 (0.76-0.80) |
| NRI (95%CI) | 0.381 (0.331-0.433) | |
| P value | <0.001 | |
| IDI (95%CI) | 0.104 (0.083-0.126) | |
| P value | <0.001 | |
| BIC | 9952.36 | 9476.02 |

CRH, cardio-renal-hepatic; NT-proBNP, N-terminal pro-B-type natriuretic peptide; NRI, net reclassification index; IDI, integrated discrimination improvement index; BIC, Bayesian information criterion; CI, confidence interval.

**Supplementary Figures.**

**
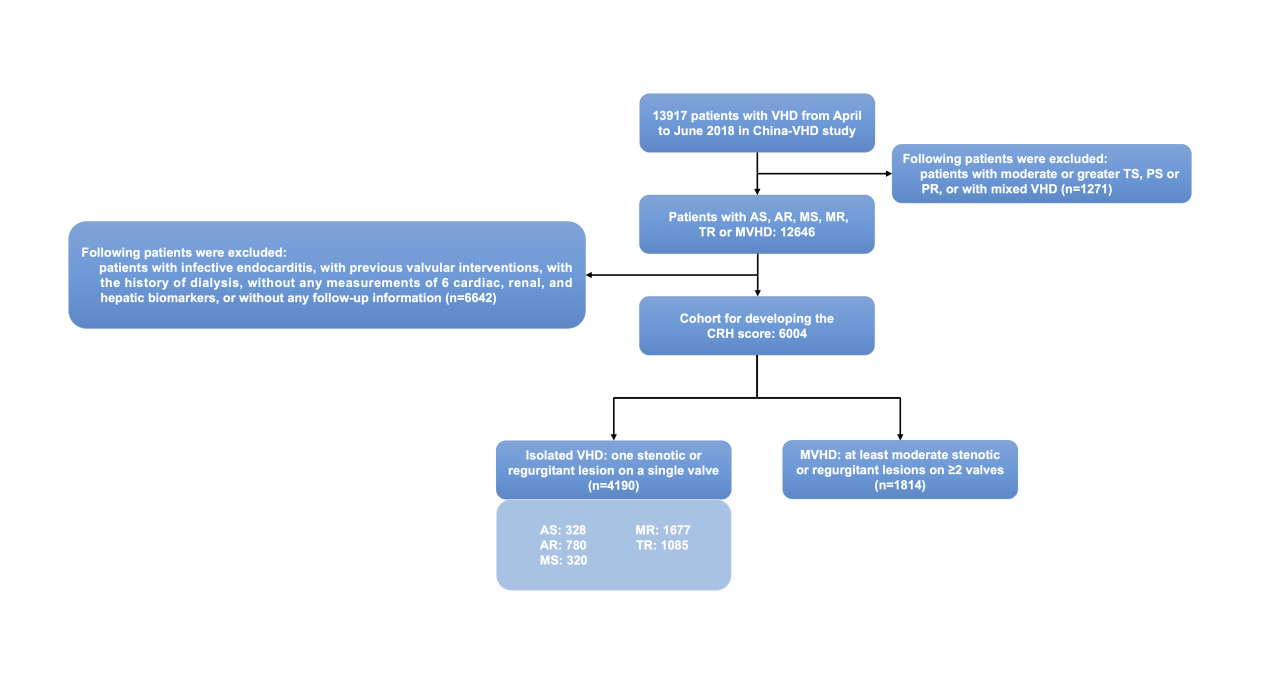
**

**Figure S1. Flowchart of the derivation cohort.** VHD, valvular heart disease; TS, tricuspid stenosis; PS, pulmonary stenosis; PR, pulmonary regurgitation; AS, aortic stenosis; AR, aortic regurgitation; MS, mitral stenosis; MR, mitral regurgitation; TR, tricuspid regurgitation; MVHD, multiple valvular heart disease; CRH, cardio-renal-hepatic.

**
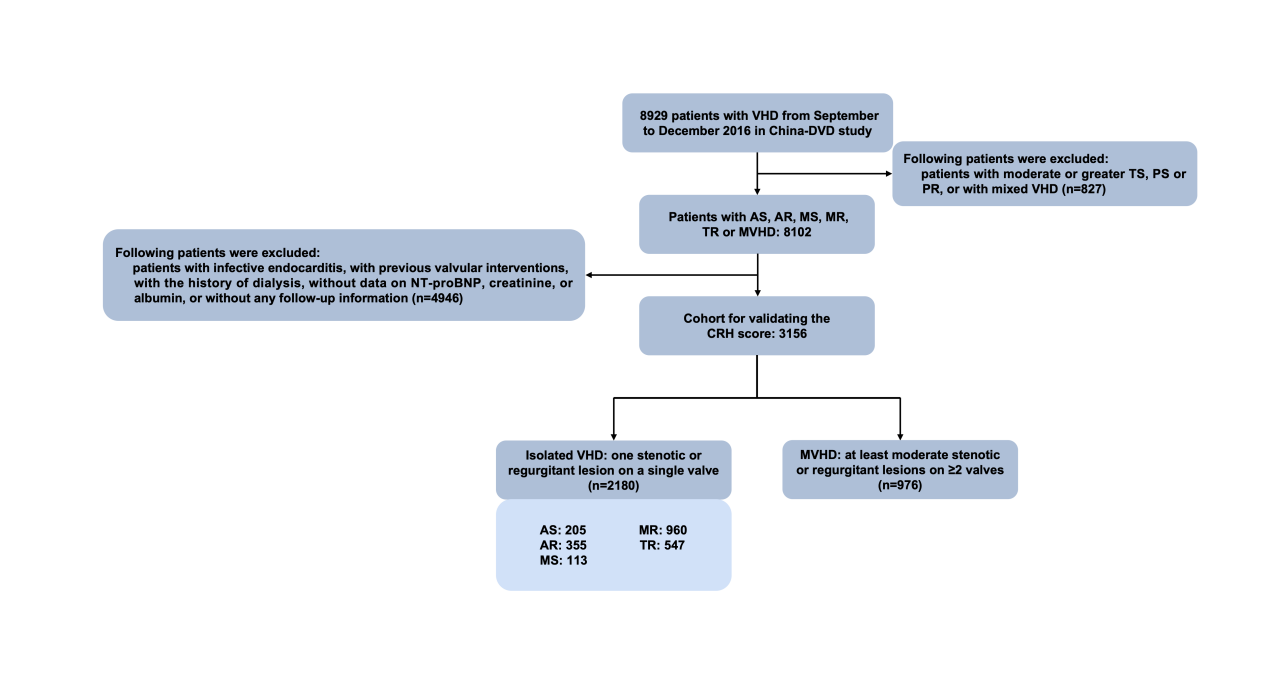
**

**Figure S2. Flowchart of the validation cohort.** VHD, valvular heart disease; China-DVD, China Elderly Valve Disease; TS, tricuspid stenosis; PS, pulmonary stenosis; PR, pulmonary regurgitation; AS, aortic stenosis; AR, aortic regurgitation; MS, mitral stenosis; MR, mitral regurgitation; TR, tricuspid regurgitation; MVHD, multiple valvular heart disease; CRH, cardio-renal-hepatic.

**
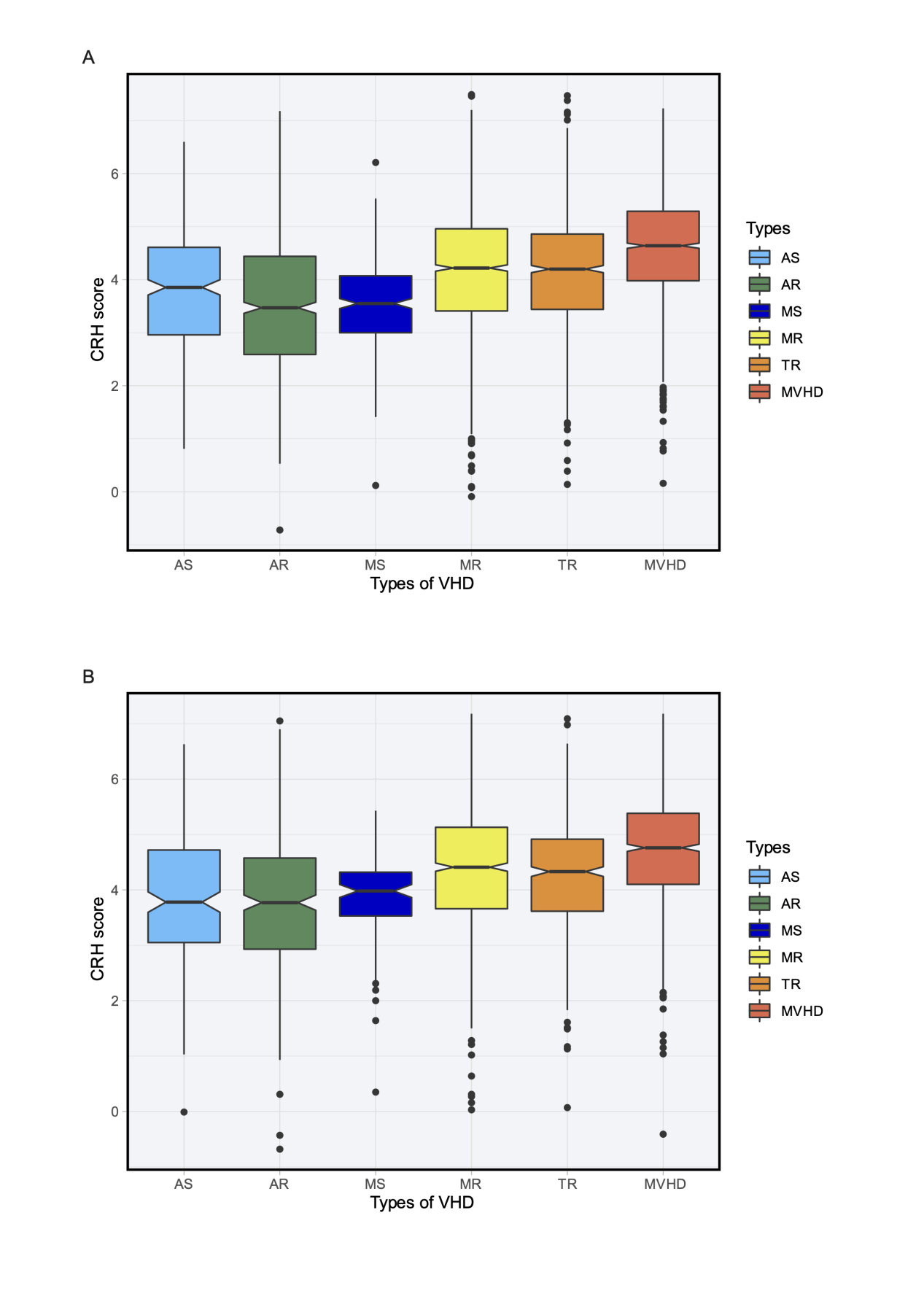
**

**Figure S3. The CRH score in different types of VHD.** (A) Distribution of the CRH score in the derivation cohort. (B) Distribution of the CRH score in the validation cohort. CRH, cardio-renal-hepatic; VHD, valvular heart disease; AS, aortic stenosis; AR, aortic regurgitation; MS, mitral stenosis; MR, mitral regurgitation; TR, tricuspid regurgitation; MVHD, multiple valvular heart disease.

**
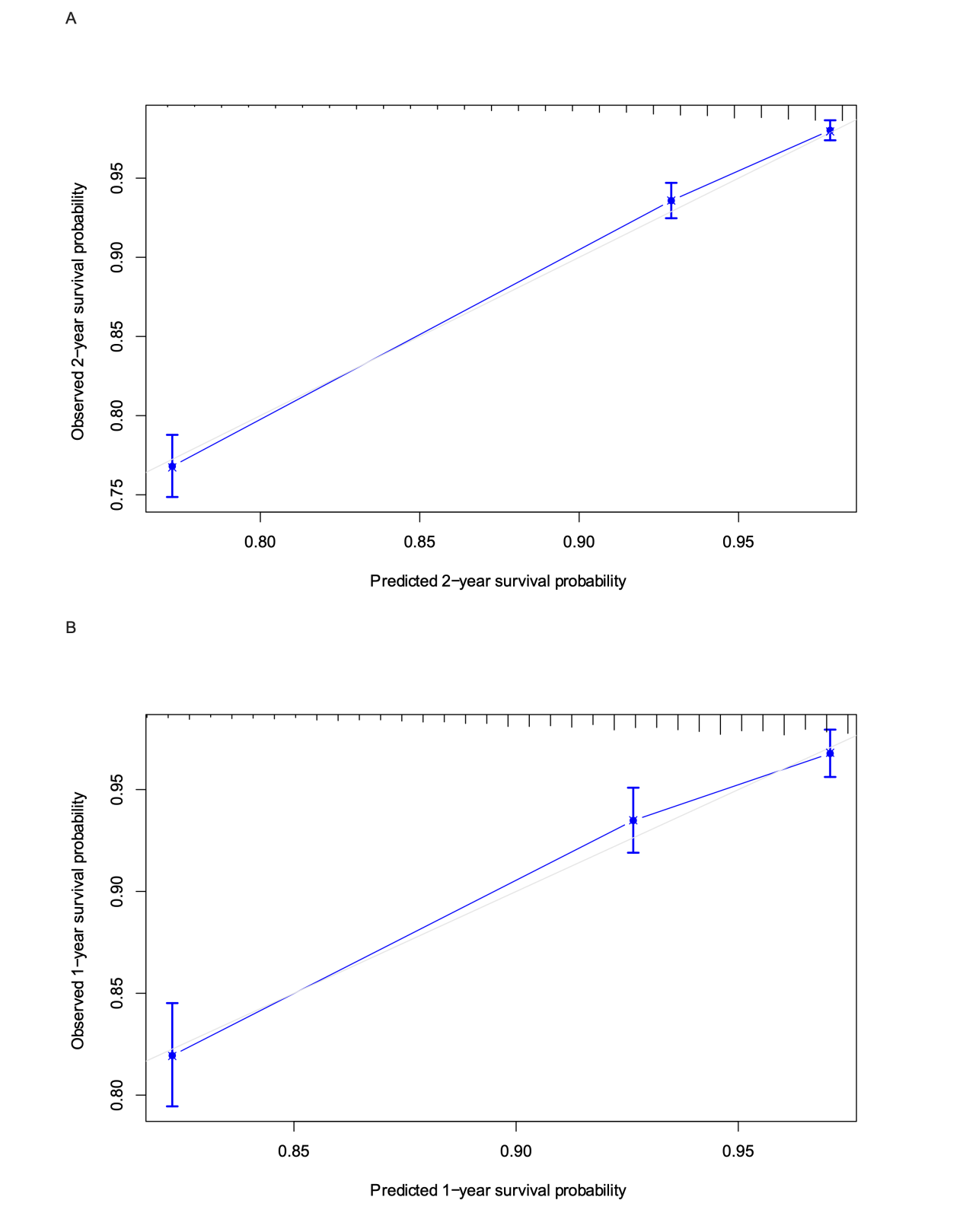
**

**Figure S4. Calibration curves of CRH score in derivation and validation cohorts.** Calibration curves present the relationship between observed and predicted survival probabilities by the CRH score. (A) Calibration curve in the derivation cohort. (B) Calibration curve in the validation cohort. CRH, cardio-renal-hepatic.

**
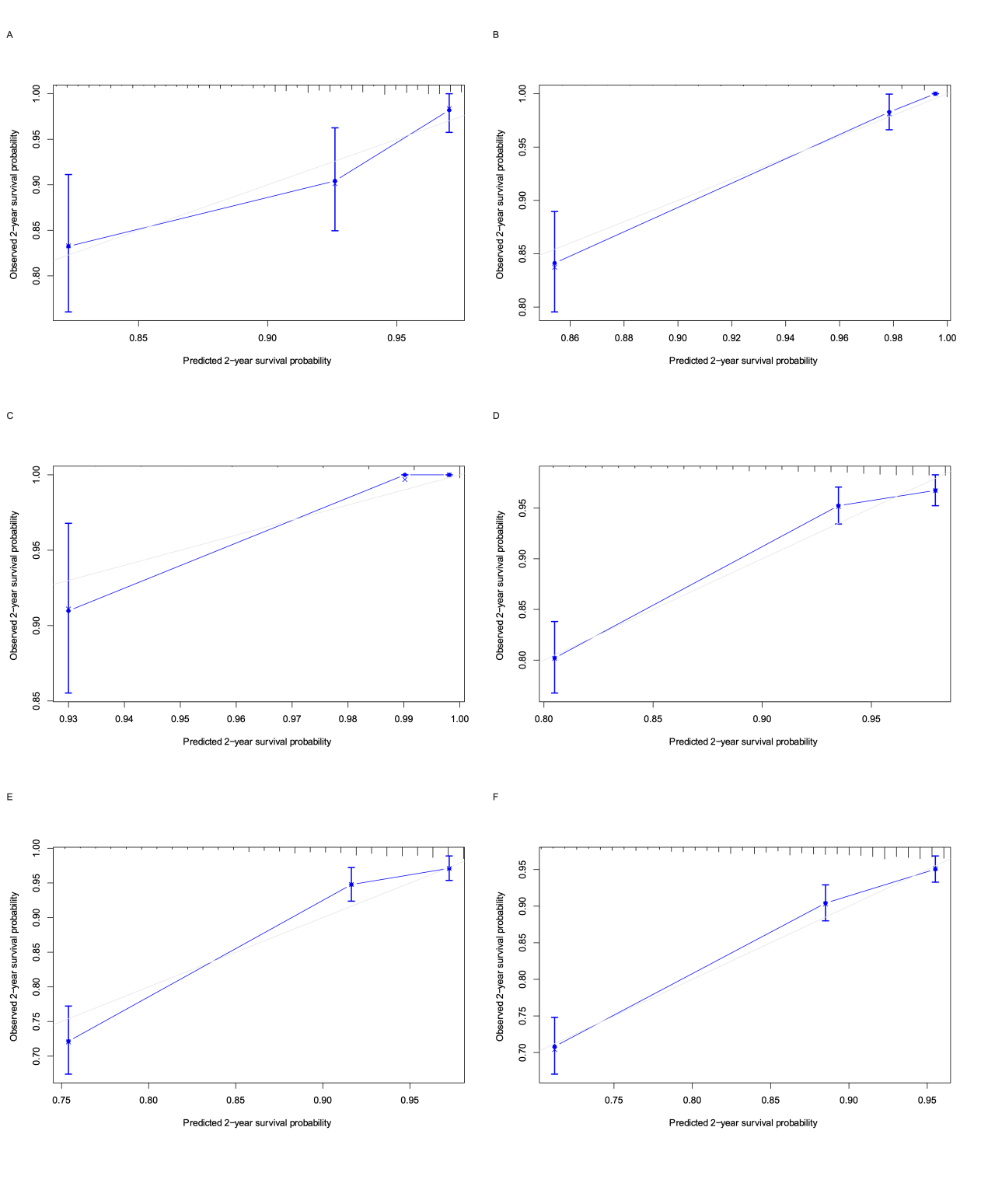
**

**Figure S5. Calibration curves of CRH score in different types of VHD in the derivation cohort.** Calibration curves present the relationship between observed and predicted survival probabilities by the CRH score. (A) Calibration curve in patients with AS. (B) Calibration curve in patients with AR. (C) Calibration curve in patients with MS. (D) Calibration curve in patients with MR. (E) Calibration curve in patients with TR. (F) Calibration curve in patients with MVHD. CRH, cardio-renal-hepatic; VHD, valvular heart disease; AS, aortic stenosis; AR, aortic regurgitation; MS, mitral stenosis; MR, mitral regurgitation; TR, tricuspid regurgitation; MVHD, multiple valvular heart disease.

**
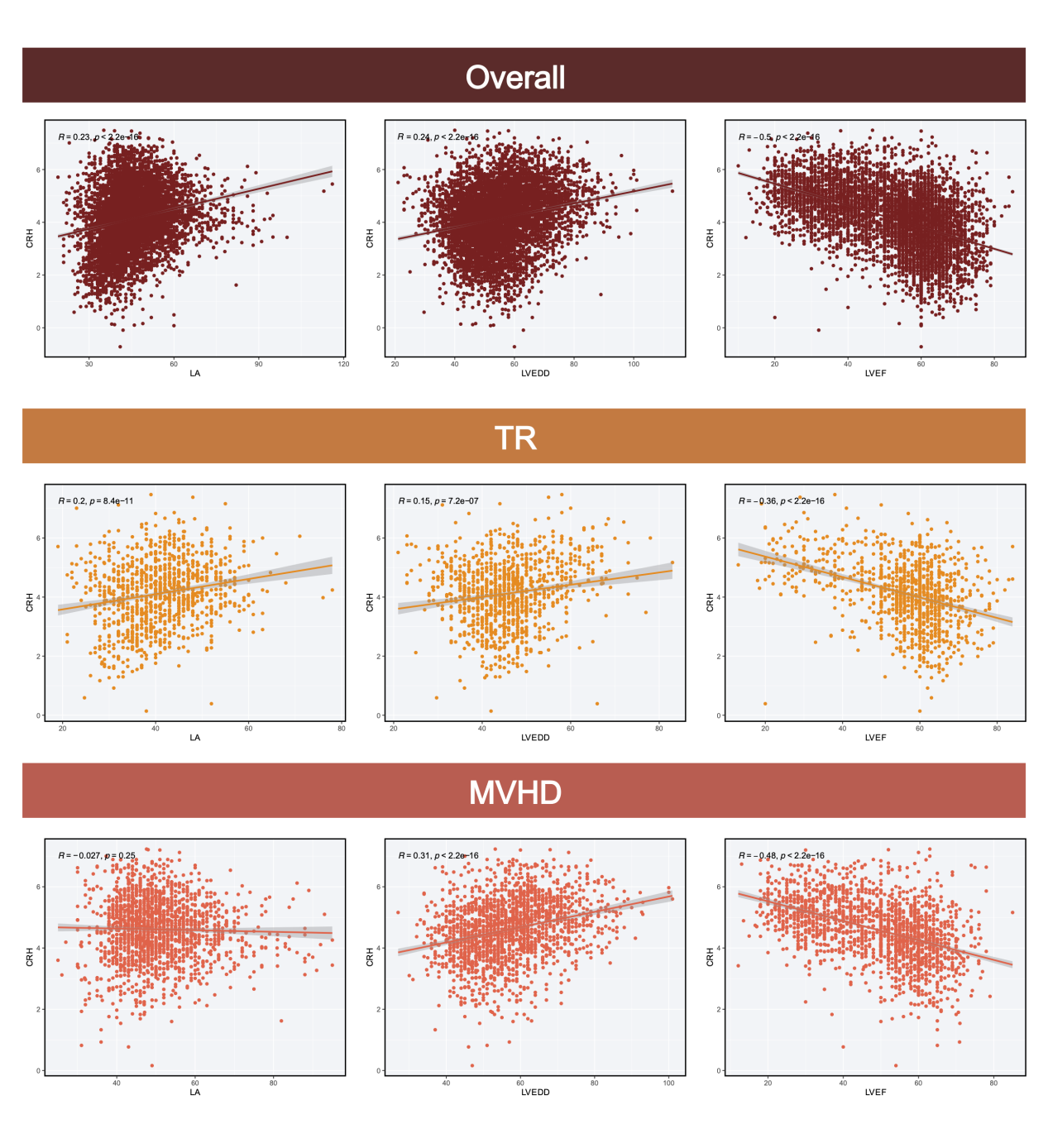
**

**Figure S6. Relationship between CRH score and echocardiographic parameters in TR and MVHD in the derivation cohort.** The spearman correlations of CRH score with LA, LVEDD, and LVEF. TR, tricuspid regurgitation; MVHD, multiple valvular heart disease; LA, left atrial end-diastolic dimension; LVEDD, left ventricular end-diastolic dimension; LVEF, left ventricular ejection fraction; CRH, cardio-renal-hepatic.

**
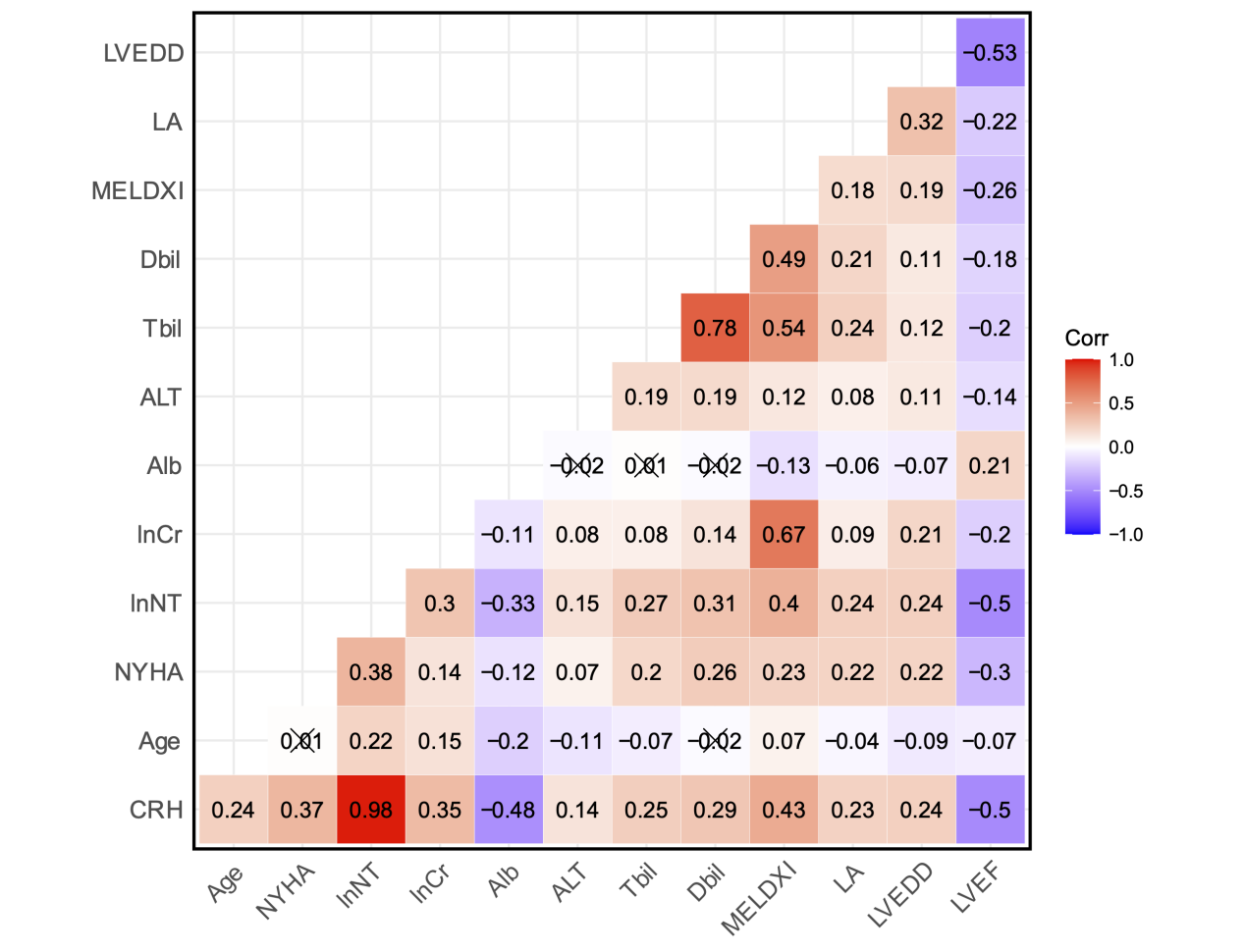
**

**Figure S7. Correlation matrix in the derivation cohort.** LVEDD, left ventricular end-diastolic dimension; LA, left atrial end-diastolic dimension; MELDXI, Model for End-stage Liver Disease excluding international normalized ratio; Dbil, direct bilirubin; Tbil, total bilirubin; ALT, alanine aminotransferase; Alb, albumin; Cr, creatinine; NT, N-terminal pro-B-type natriuretic peptide; NYHA, New York Heart Association; CRH, cardio-renal-hepatic.

**
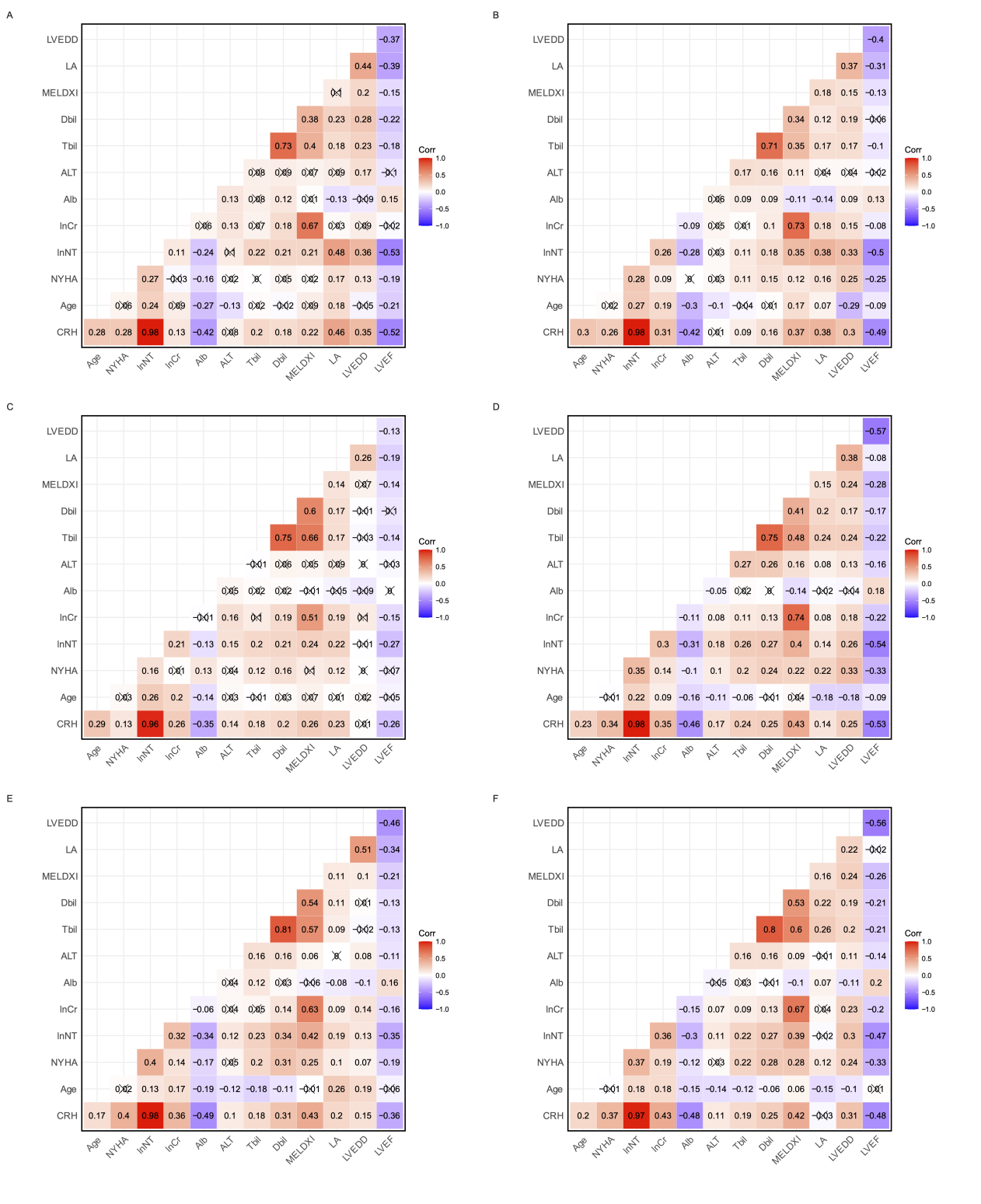
**

**Figure S8. Correlation matrices in different types of VHD.** (A) Correlation matrix in AS. (B) Correlation matrix in AR. (C) Correlation matrix in MS. (D) Correlation matrix in MR. (E) Correlation matrix in TR. (F) Correlation matrix in MVHD. LVEDD, left ventricular end-diastolic dimension; LA, left atrial end-diastolic dimension; MELDXI, Model for End-stage Liver Disease excluding international normalized ratio; Dbil, direct bilirubin; Tbil, total bilirubin; ALT, alanine aminotransferase; Alb, albumin; Cr, creatinine; NT, N-terminal pro-B-type natriuretic peptide; NYHA, New York Heart Association; CRH, cardio-renal-hepatic; VHD, valvular heart disease; AS, aortic stenosis; AR, aortic regurgitation; MS, mitral stenosis; MR, mitral regurgitation; TR, tricuspid regurgitation; MVHD, multiple valvular heart disease.

**
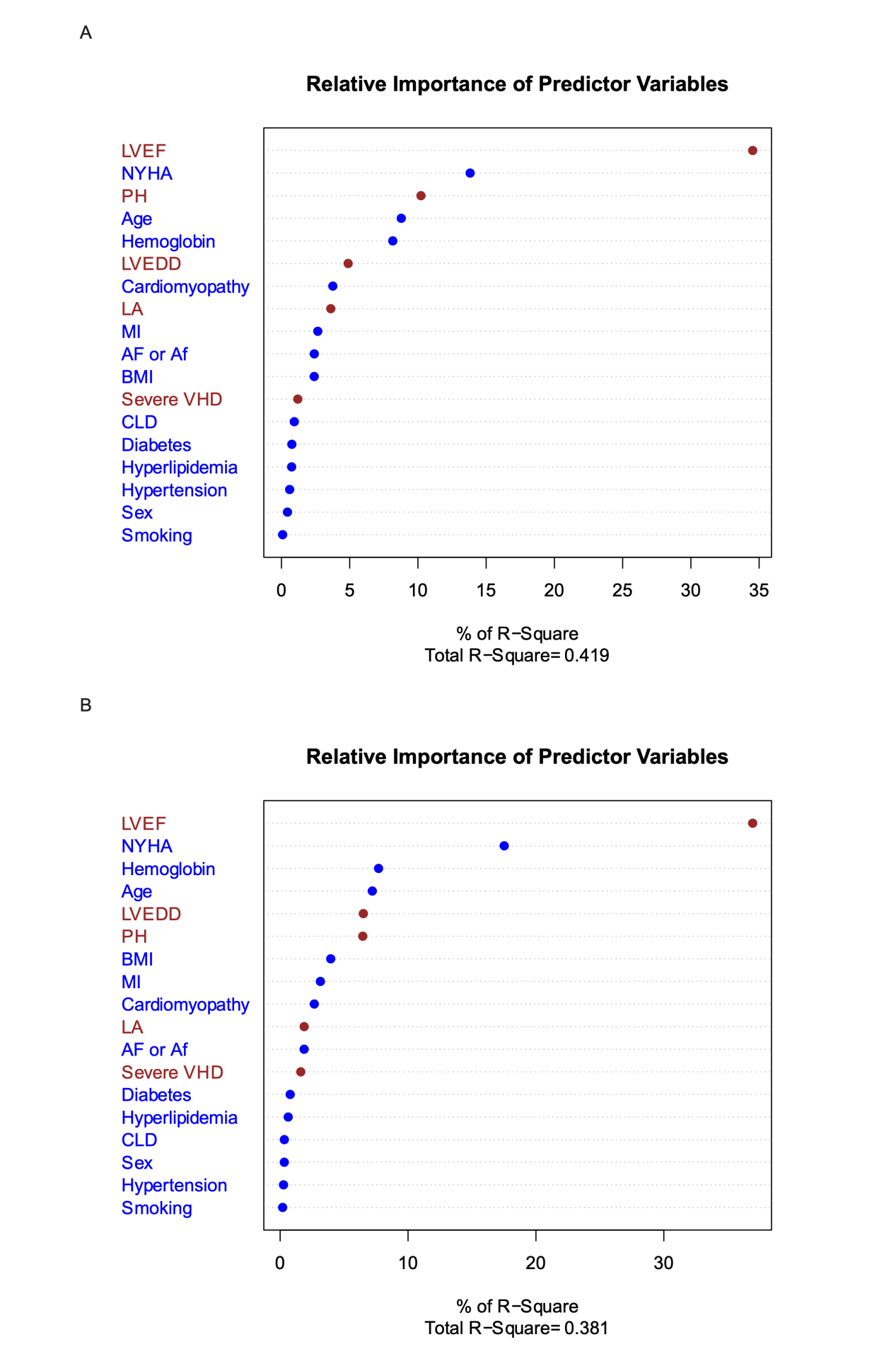
**

**Figure S9. Relative importance of predictors of cardio-renal-hepatic co-dysfunction in derivation and validation cohorts.** Variable importance was evaluated and ranked by the relative weight of predictors, which was defined as the contribution each predictor made to total R^2^. (A) Relative importance of predictors in the derivation cohort. (B) Relative importance of predictors in the validation cohort. LVEF, left ventricular ejection fraction; NYHA, New York Heart Association; PH, pulmonary hypertension; LVEDD, left ventricular end-diastolic dimension; LA, left atrial end-diastolic dimension; MI, myocardial infarction; AF, atrial fibrillation; Af, atrial flutter; BMI, body mass index; VHD, valvular heart disease; CLD, chronic lung disease.

**
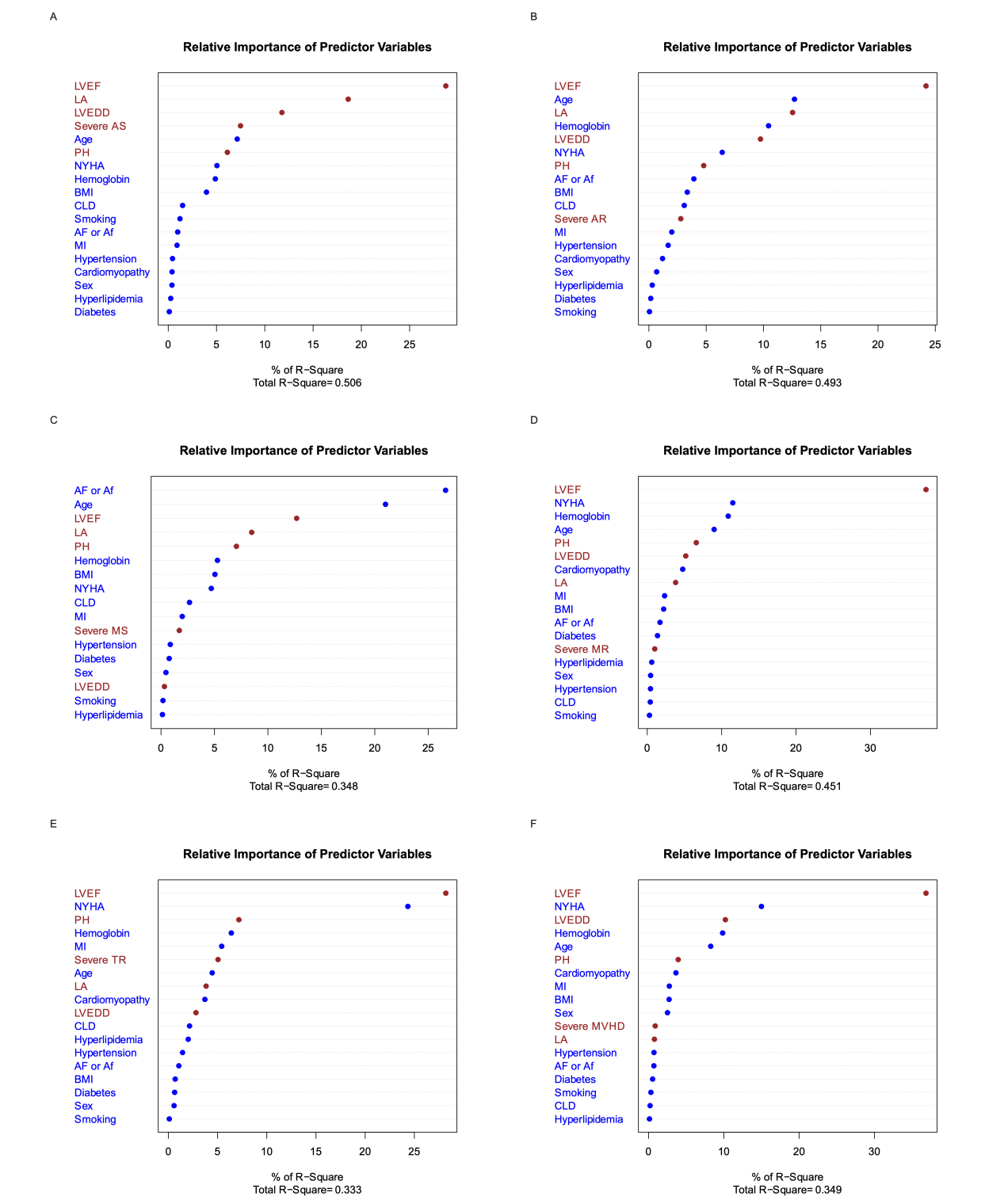
**

**Figure S10.** **Relative importance of predictors of cardio-renal-hepatic co-dysfunction in different types of VHD in derivation cohort.** Variable importance was evaluated and ranked by the relative weight of predictors, which was defined as the contribution each predictor made to total R^2^. (A) Relative importance of predictors in AS. (B) Relative importance of predictors in AR. (C) Relative importance of predictors in MS. (D) Relative importance of predictors in MR. (E) Relative importance of predictors in TR. (F) Relative importance of predictors in MVHD. LVEF, left ventricular ejection fraction; LA, left atrial end-diastolic dimension; LVEDD, left ventricular end-diastolic dimension; PH, pulmonary hypertension; NYHA, New York Heart Association; BMI, body mass index; CLD, chronic lung disease; AF, atrial fibrillation; Af, atrial flutter; MI, myocardial infarction; VHD, valvular heart disease; AS, aortic stenosis; AR, aortic regurgitation; MS, mitral stenosis; MR, mitral regurgitation; TR, tricuspid regurgitation; MVHD, multiple valvular heart disease.

**
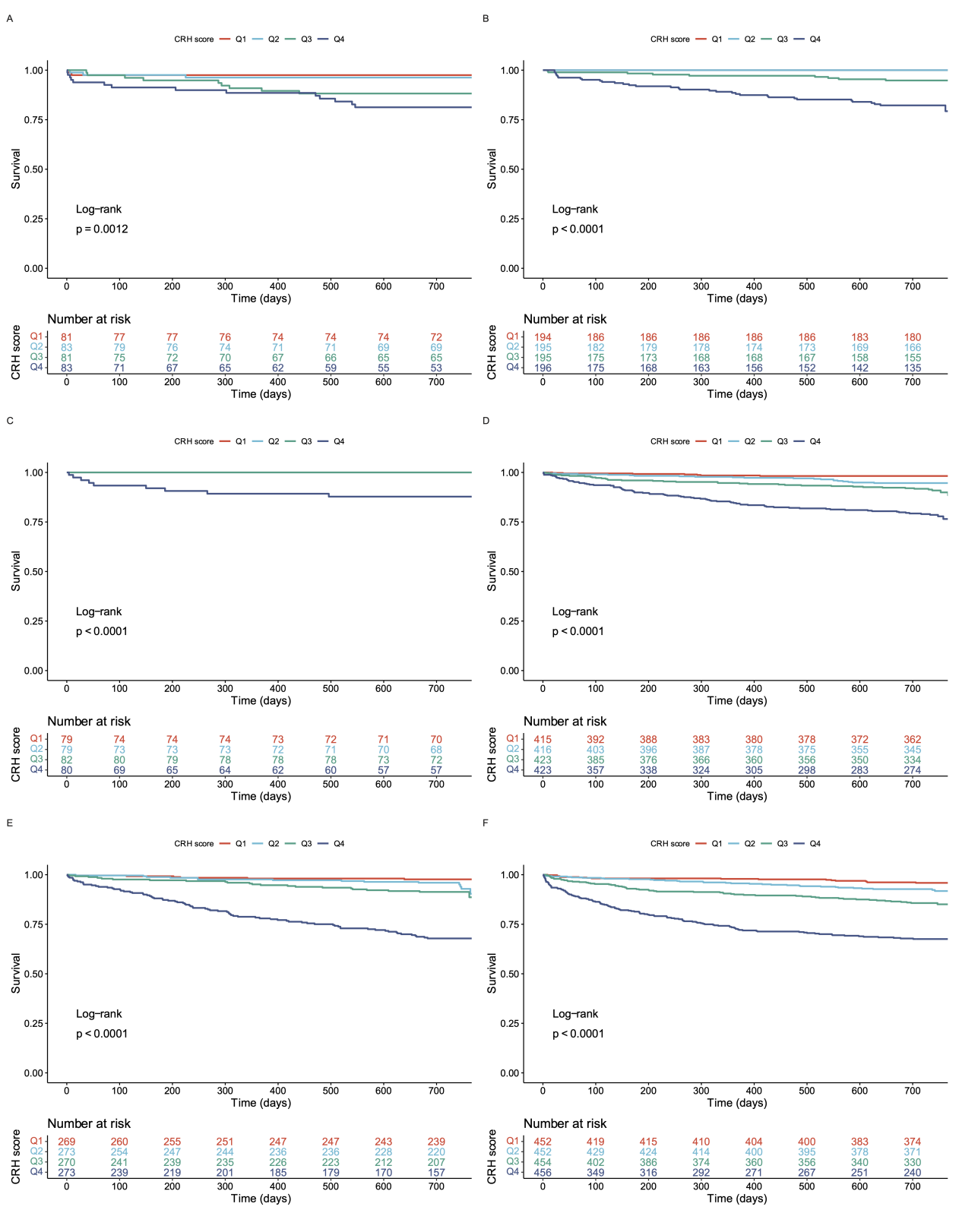
**

**Figure S11. Kaplan-Meier curves according to types of VHD in the derivation cohort.** (A) Kaplan-Meier curve in AS. (B) Kaplan-Meier curve in AR. (C) Kaplan-Meier curve in MS. (D) Kaplan-Meier curve in MR. (E) Kaplan-Meier curve in TR. (F) Kaplan-Meier curve in MVHD. CRH, cardio-renal-hepatic; VHD, valvular heart disease; AS, aortic stenosis; AR, aortic regurgitation; MS, mitral stenosis; MR, mitral regurgitation; TR, tricuspid regurgitation; MVHD, multiple valvular heart disease.


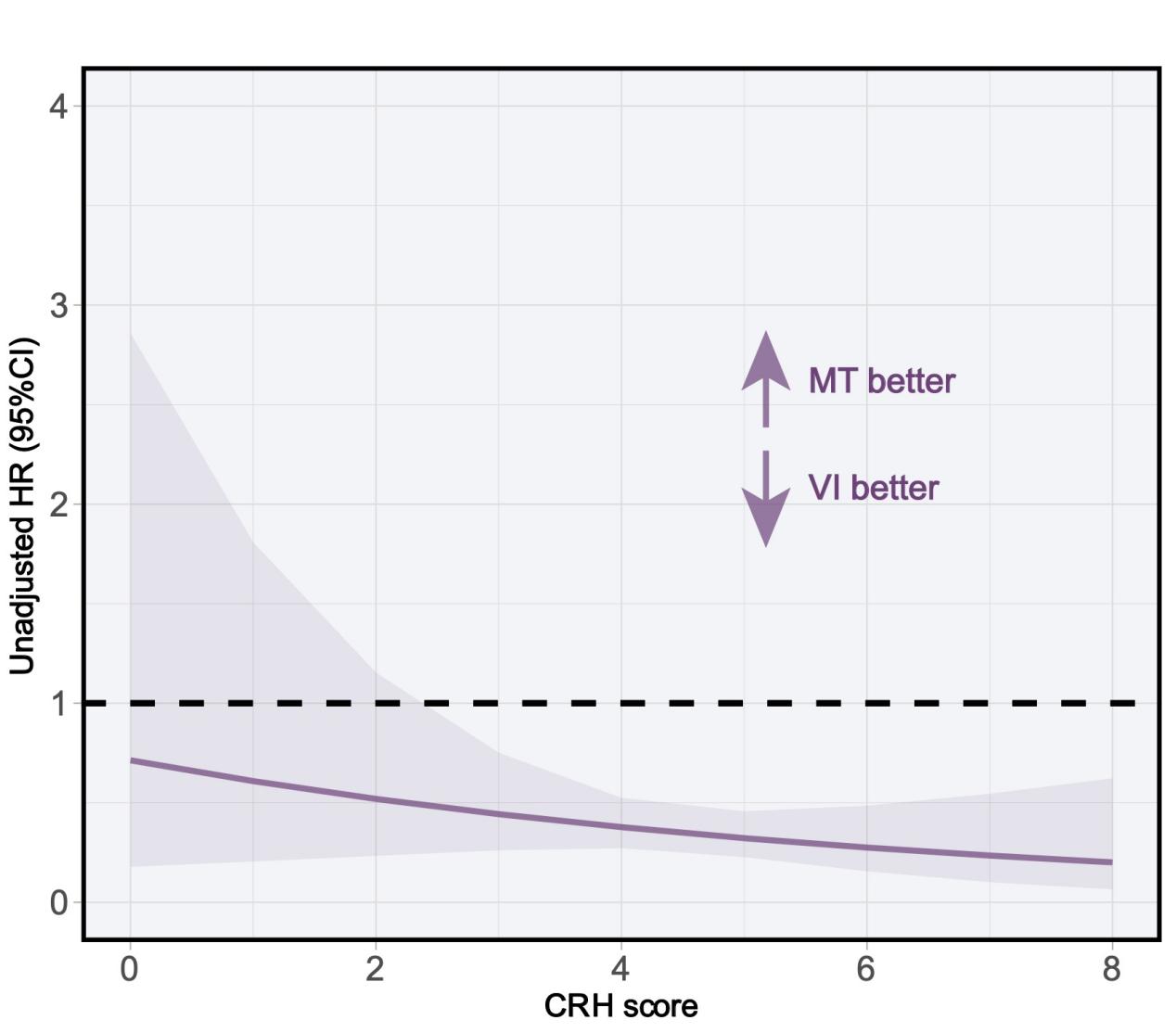


**Figure S12. Survival benefit of VI beyond MT according to CRH score.** CRH, cardio-renal-hepatic; MT, medial treatment; VI, valvular intervention; HR, hazard ratio.

**
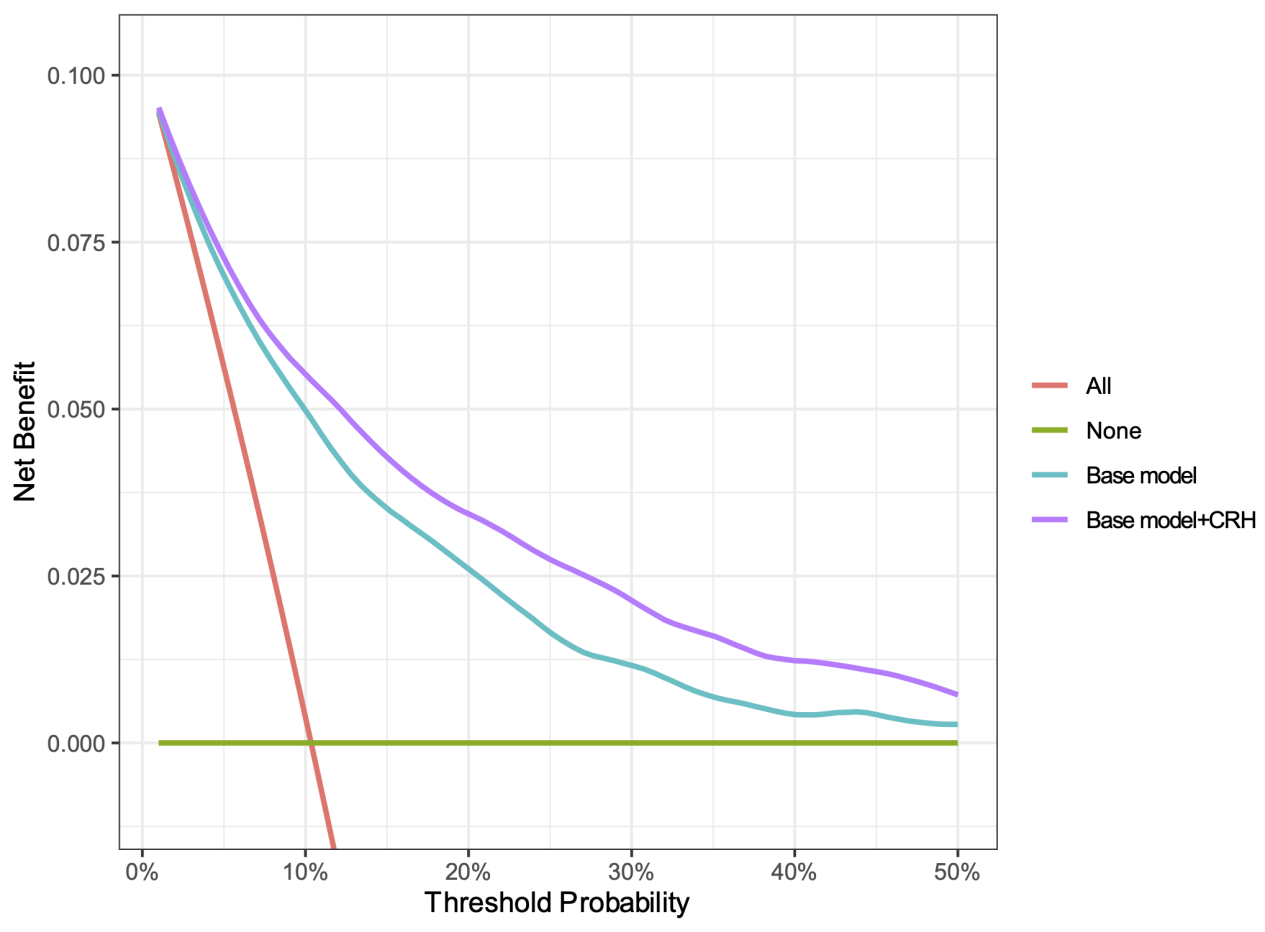
**

**Figure S13. Decision curve analysis in the derivation cohort.** The red and green solid lines represented the assumption that all or none patients at high risk with various thresholds. The lines in the upper right represented different prognostic models. CRH, cardio-renal-hepatic.

**
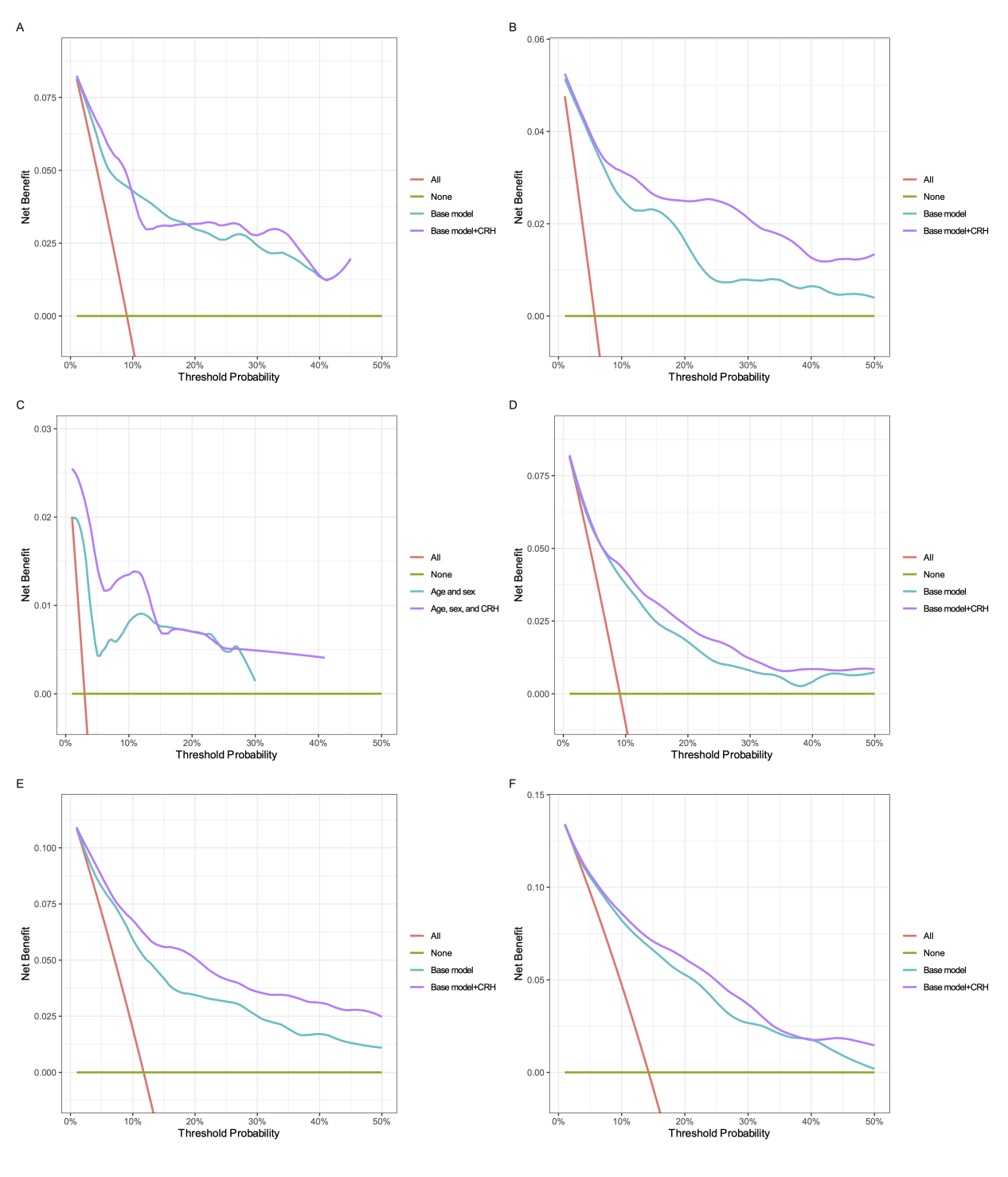
**

**Figure S14. Decision curve analysis in different types of VHD.** The red and green solid lines represented the assumption that all or none patients at high risk with various thresholds. The lines in the upper right represented different prognostic models. (A) Decision curve analysis in AS. (B) Decision curve analysis in AR. (C) Decision curve analysis in MS. (D) Decision curve analysis in MR. (E) Decision curve analysis in TR. (F) Decision curve analysis in MVHD. CRH, cardio-renal-hepatic; VHD, valvular heart disease; AS, aortic stenosis; AR, aortic regurgitation; MS, mitral stenosis; MR, mitral regurgitation; TR, tricuspid regurgitation; MVHD, multiple valvular heart disease.

**
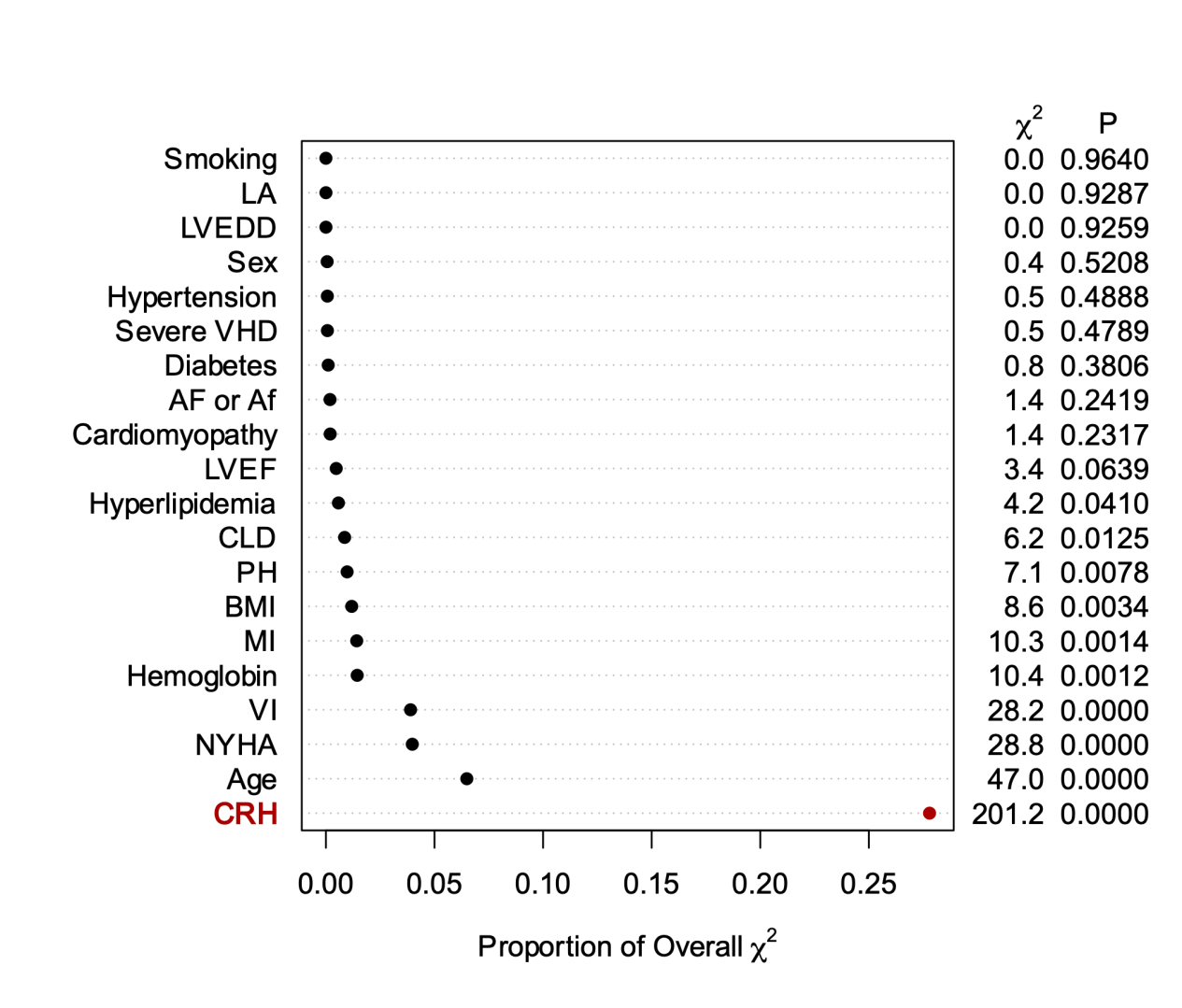
**

**Figure S15. Relative importance of predictors by the proportion of explainable log-likelihood ratio χ^2^ statistics in the derivation cohort.** Relative importance of variables was ranked by the proportion of explainable log-likelihood ratio χ^2^ statistics. LA, left atrial end-diastolic dimension; LVEDD, left ventricular end-diastolic dimension; VHD, valvular heart disease; AF, atrial fibrillation; Af, atrial flutter; LVEF, left ventricular ejection fraction; CLD, chronic lung disease; PH, pulmonary hypertension; BMI, body mass index; MI, myocardial infarction; VI, valvular intervention; NYHA, New York Heart Association; CRH, cardio-renal-hepatic.

**
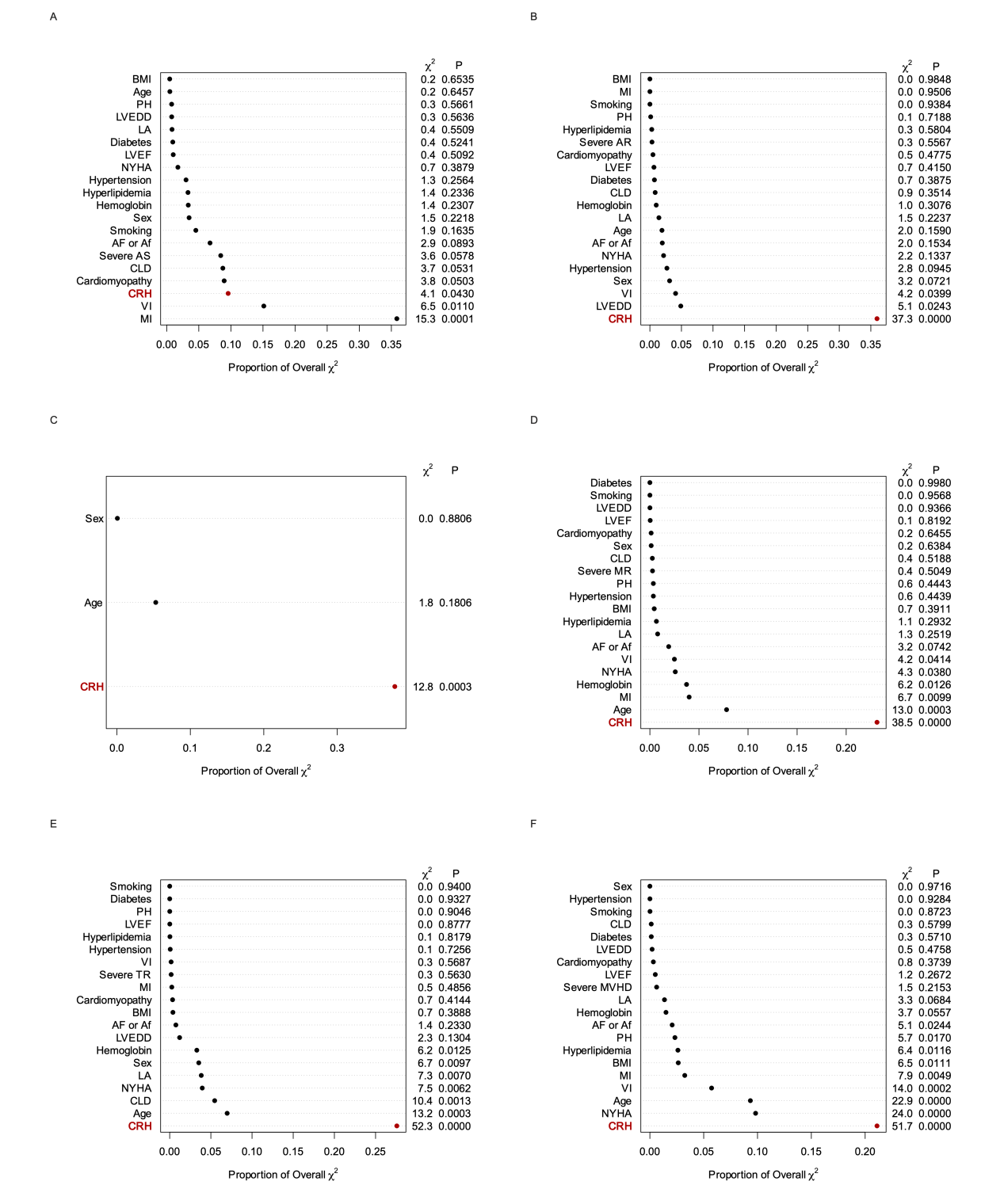
**

**Figure S16. Relative importance of predictors by the proportion of explainable log-likelihood ratio χ^2^ statistics in different types of VHD.** Relative importance of variables was ranked by the proportion of explainable log-likelihood ratio χ^2^ statistics. (A) Relative importance of predictors in AS. (B) Relative importance of predictors in AR. (C) Relative importance of predictors in MS. (D) Relative importance of predictors in MR. (E) Relative importance of predictors in TR. (F) Relative importance of predictors in MVHD. BMI, body mass index; PH, pulmonary hypertension; LVEDD, left ventricular end-diastolic dimension; LA, left atrial end-diastolic dimension; LVEF, left ventricular ejection fraction; NYHA, New York Heart Association; AF, atrial fibrillation; Af, atrial flutter; CLD, chronic lung disease; CRH, cardio-renal-hepatic; VI, valvular intervention; MI, myocardial infarction; VHD, valvular heart disease; AS, aortic stenosis; AR, aortic regurgitation; MS, mitral stenosis; MR, mitral regurgitation; TR, tricuspid regurgitation; MVHD, multiple valvular heart disease.

**
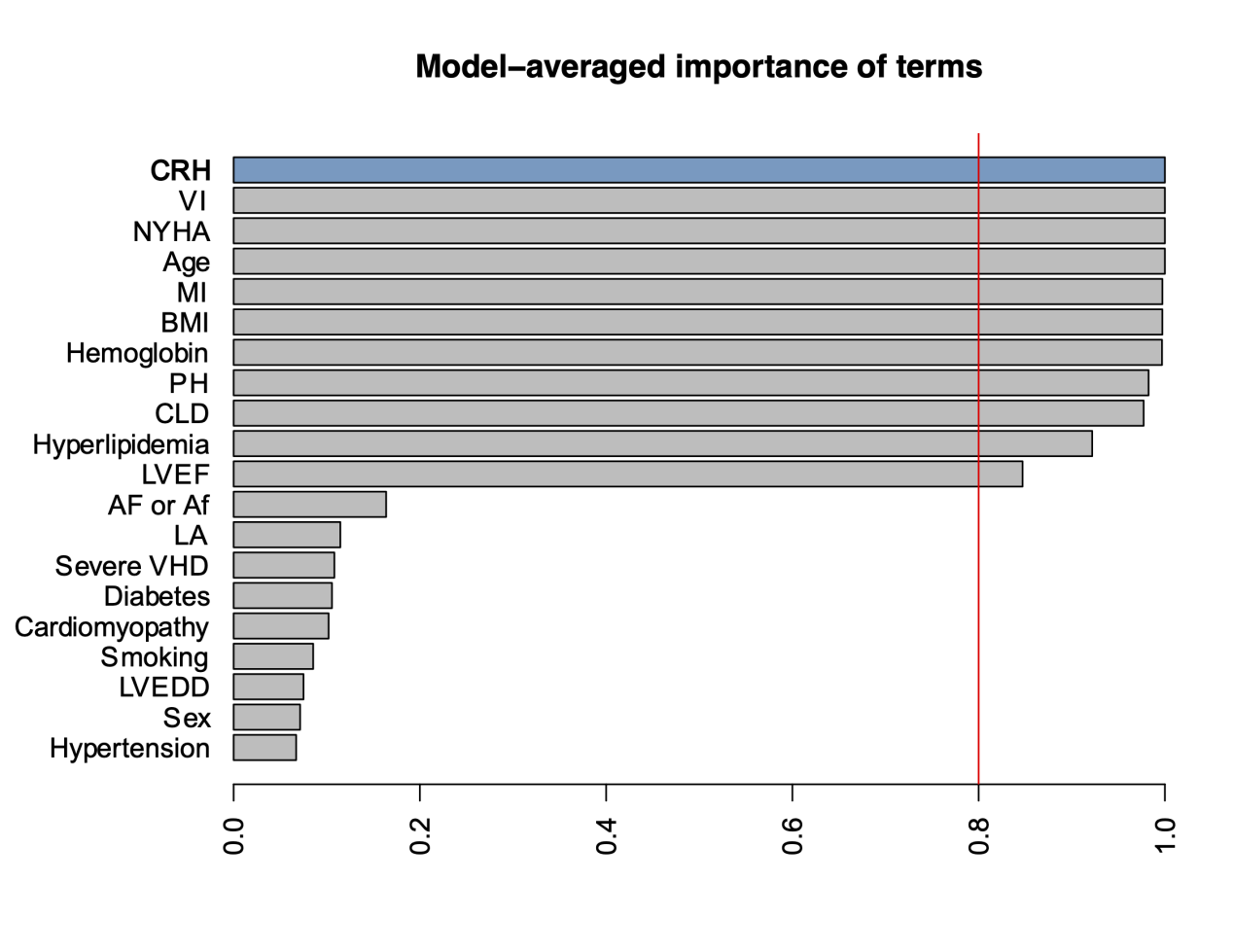
**

Best IC: 9261.27204210414

Worst IC: 9285.01935799724

11 models within 2 IC units.

20 models to reach 95% of evidence weight.

100 best models from 1180 generations.

**Best model: CRH, age, BMI, hyperlipidemia, MI, CLD, NYHA, hemoglobin, LVEF, PH, VI**

**Figure S17. Relative importance of predictors by best subset analysis in the derivation cohort.** Model-averaged importance of predictors was determined by finding 100 best models among the possible models. Models were ranked by Akaike Information Criterion, and the best models were found by a genetic algorithm. CRH, cardio-renal-hepatic; VI, valvular intervention; NYHA, New York Heart Association; MI, myocardial infarction; BMI, body mass index; PH, pulmonary hypertension; CLD, chronic lung disease; LVEF, left ventricular ejection fraction; AF, atrial fibrillation; Af, atrial flutter; LA, left atrial end-diastolic dimension; VHD, valvular heart disease; LVEDD, left ventricular end-diastolic dimension.

**
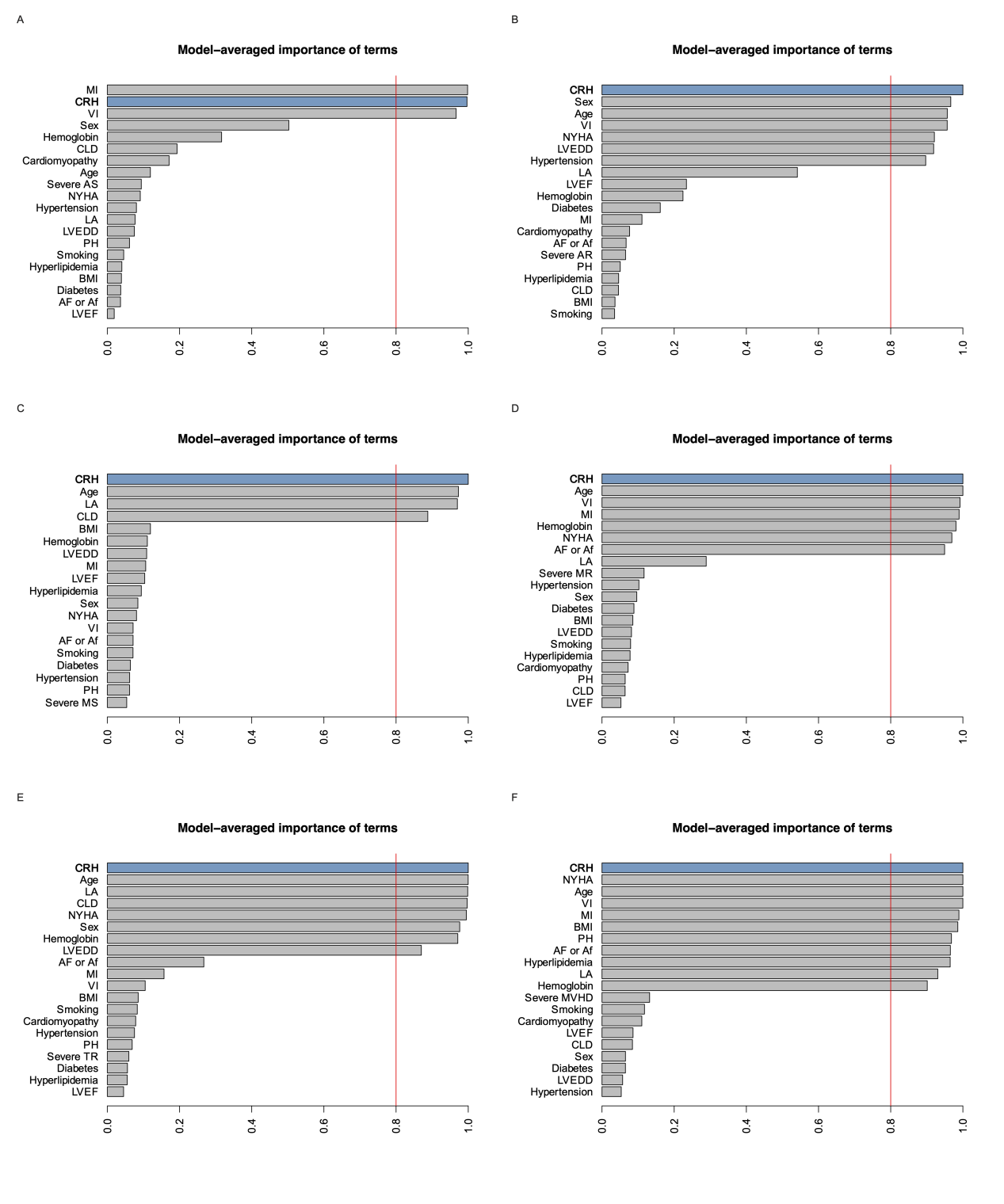
**

Best IC: 3259.49677209523

Worst IC: 3279.77080325891

11 models within 2 IC units.

18 models to reach 95% of evidence weight.

100 best models from 630 generations.

**Best model: CRH, age, BMI, hyperlipidemia, MI, AF or Af, NYHA, hemoglobin, LA, PH, VI**

Best IC: 1498.30043682752

Worst IC: 1512.58453190366

14 models within 2 IC units.

25 models to reach 95% of evidence weight.

100 best models from 870 generations.

**Best model: CRH, age, sex, CLD, NYHA, hemoglobin, LA, LVEDD**

Best IC: 73.1846983699768

Worst IC: 84.1801638657656

17 models within 2 IC units.

37 models to reach 95% of evidence weight.

100 best models from 300 generations.

**Best model: CRH, age, CLD, LA**

Best IC: 1946.97646076037

Worst IC: 1960.22537246389

14 models within 2 IC units.

26 models to reach 95% of evidence weight.

100 best models from 480 generations.

**Best model: CRH, age, MI, AF or Af, NYHA, hemoglobin, VI**

Best IC: 294.256052107916

Worst IC: 304.697057803117

24 models within 2 IC units.

43 models to reach 95% of evidence weight.

100 best models from 340 generations.

**Best model: CRH, sex, MI, VI**

Best IC: 434.863470412898

Worst IC: 444.847880968498

20 models within 2 IC units.

47 models to reach 95% of evidence weight.

100 best models from 420 generations.

**Best model: CRH, age, sex, Hypertension, NYHA, LA, LVEDD, VI**

**Figure S18. Relative importance of predictors by best subset analysis in different types of VHD.** Model-averaged importance of predictors was determined by finding 100 best models among the possible models. Models were ranked by Akaike Information Criterion, and the best models were found by a genetic algorithm. (A) Relative importance of predictors in AS. (B) Relative importance of predictors in AR. (C) Relative importance of predictors in MS. (D) Relative importance of predictors in MR. (E) Relative importance of predictors in TR. (F) Relative importance of predictors in MVHD. MI, myocardial infarction; CRH, cardio-renal-hepatic; VI, valvular intervention; CLD, chronic lung disease; NYHA, New York Heart Association; LA, left atrial end-diastolic dimension; LVEDD, left ventricular end-diastolic dimension; PH, pulmonary hypertension; BMI, body mass index; AF, atrial fibrillation; Af, atrial flutter; LVEF, left ventricular ejection fraction. VHD, ; AS, aortic stenosis; AR, aortic regurgitation; MS, mitral stenosis; MR, mitral regurgitation; TR, tricuspid regurgitation; MVHD, multiple valvular heart disease.

**
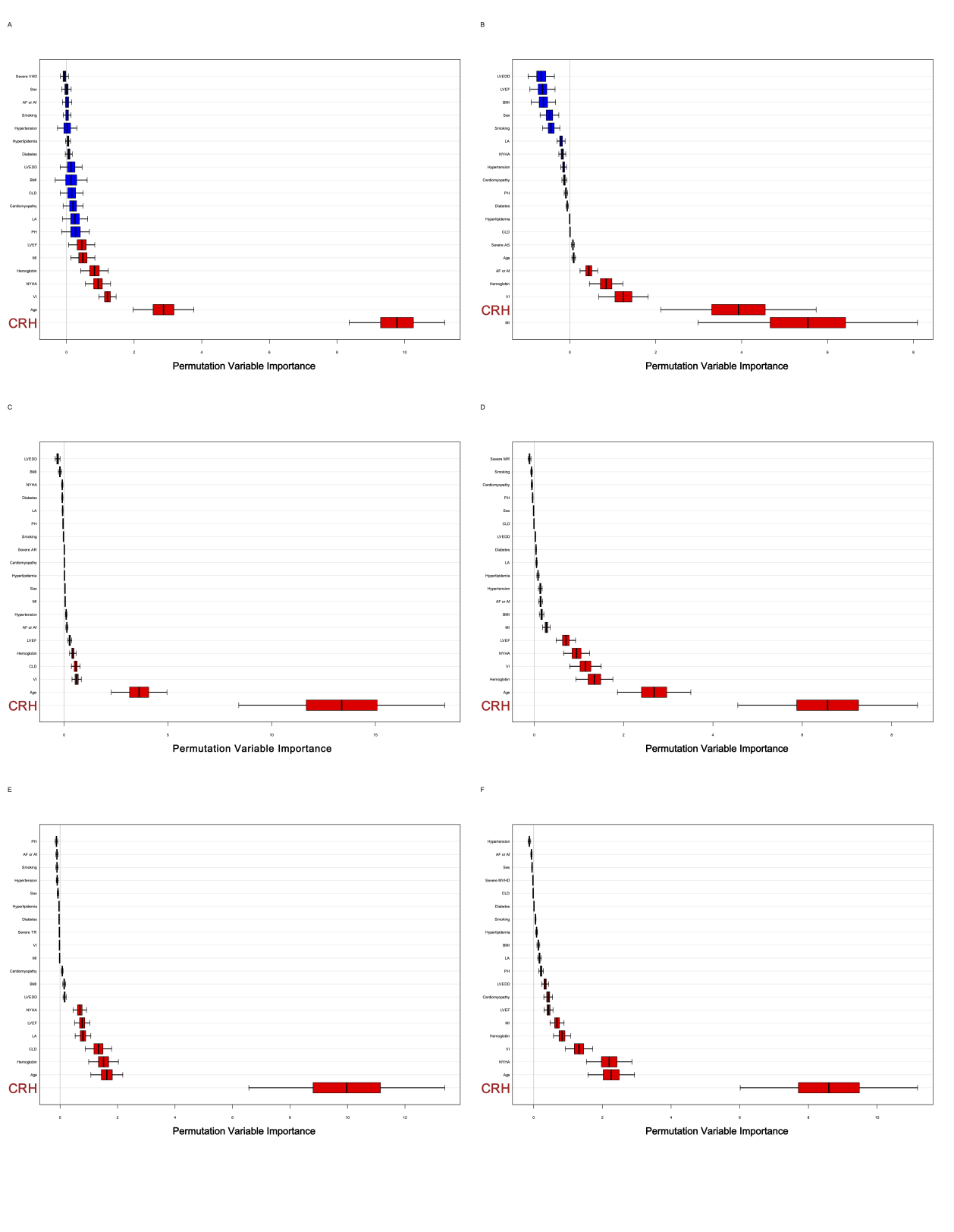
**

**Figure S19. Variable importance estimated by random survival forest.** (A) Variable importance in the derivation cohort. (B) Variable importance in AS. (C) Variable importance in AR. (D) Variable importance in MR. (E) Variable importance in TR. (F) Variable importance in MVHD.

**
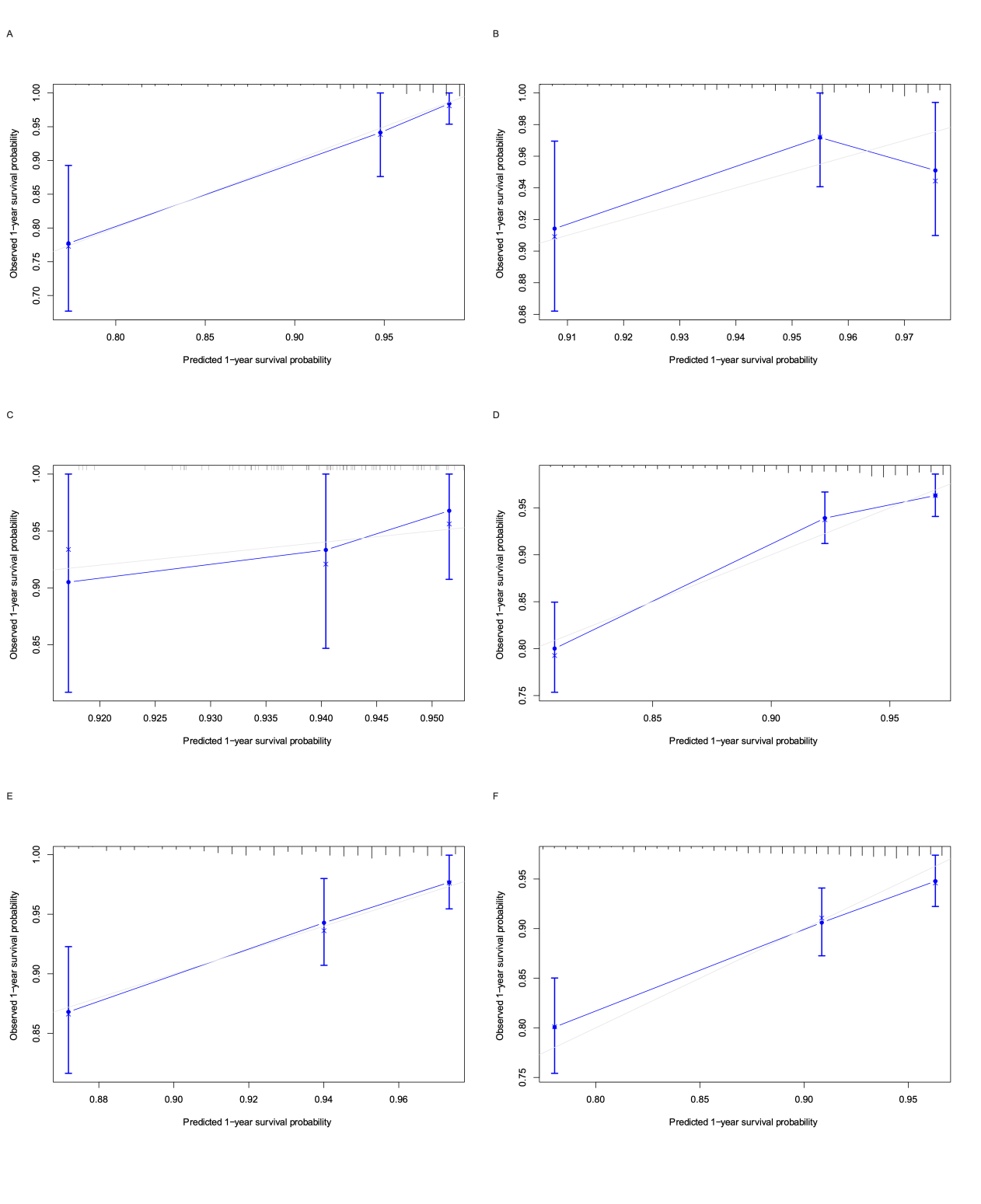
**

**Figure S20. Calibration curves of CRH score in different types of VHD in the validation cohort.** Calibration curves present the relationship between observed and predicted survival probabilities by the CRH score. (A) Calibration curve in patients with AS. (B) Calibration curve in patients with AR. (C) Calibration curve in patients with MS. (D) Calibration curve in patients with MR. (E) Calibration curve in patients with TR. (F) Calibration curve in patients with MVHD. CRH, cardio-renal-hepatic; VHD, valvular heart disease; AS, aortic stenosis; AR, aortic regurgitation; MS, mitral stenosis; MR, mitral regurgitation; TR, tricuspid regurgitation; MVHD, multiple valvular heart disease.
