# Supplementary material for: Assessment of cardio-renal-hepatic function in patients with valvular heart disease: a multi-biomarker approach—the cardio-renal-hepatic score
Source: BMC Med. 2023 Jul 17;21:257. doi: 10.1186/s12916-023-02971-y (PMC10351119; doi:10.1186/s12916-023-02971-y)
Supplement: Supplementary file 2 — Additional file 2: Page S1. Echocardiographic criteria of significant VHD in the China-VHD study. Page S1. Statistical analysis. [file 12916_2023_2971_MOESM2_ESM.docx]

**Echocardiographic criteria of significant VHD in the China-VHD study**

**Significant AR:** Criteria for significant AR were as follows: jet width ≥25% of LV outflow tract, or regurgitant volume ≥30mL/beat, or vena contracta ≥0.3cm, or regurgitant fraction ≥30%, or effective regurgitation orifice ≥0.10cm^2^. Criteria for severe AR were as follows: jet width ≥65% of LV outflow tract, or regurgitant volume ≥60mL/beat, or vena contracta ≥0.6cm, or regurgitant fraction ≥50%, or effective regurgitation orifice ≥0.30cm^2^, or regurgitant jet reaching the level of left ventricular chordae tendineae or apex, or holodiastolic flow reversal in descending aorta.

**Significant AS:** Criteria for significant AS were as follows: a valve area ≤1.5cm^2^, or a maximal jet velocity ≥3m/s, or a mean pressure gradient ≥20mmHg. Criteria for severe AS were as follows: a valve area ≤1.0cm^2^, or a maximal jet velocity ≥4m/s, or a mean pressure gradient ≥40mmHg.

**Significant MR:** Criteria for significant MR were as follows: vena contracta ≥0.3cm, or regurgitant volume ≥30mL/beat, or central jet MR>20% left atrium, or effective regurgitation orifice ≥0.20cm^2^. Criteria for severe MR were as follows: vena contracta ≥0.7cm, or regurgitant volume ≥60mL/beat (for primary MR) or ≥30mL/beat (for secondary MR), or central jet MR>40% left atrium, or effective regurgitation orifice ≥0.40cm^2^ (for primary MR) or ≥0.20cm^2^ (for secondary MR), or regurgitant jet reaching the posterior wall or the top of the left atrium.

**Significant MS:** The criterion for significant MS was a valve area ≤1.5cm^2^. The criterion for severe MS was a valve area ≤1.0cm^2^.

**Significant TR:** Regurgitation with moderate or more severe grade or central jet area >5 cm^2^ at tricuspid lesion was identified as significant TR. Criteria for severe TR were as follows: central jet area >10 cm^2^, or vena contracta width >0.7 cm, or regurgitant volume ≥45 ml/beat, or effective regurgitant orifice area ≥40 mm^2^, or central jet TR >2/3 right atrium, or flow reversal to the top of right atrium or in the inferior vena cava.

**Significant MVHD:** Significant MVHD was the combination of moderate or greater regurgitant or stenotic lesions on at least two valves. Severe MVHD was defined as MVHD patients with at least one severe valvular lesion.

**Statistical analysis**

Multiple linear regression models were used to explore the associated factors of the CRH score, with variables including age, sex, BMI, smoking status, hypertension, hyperlipidemia, diabetes, prior myocardial infarction, cardiomyopathy, atrial fibrillation or flutter, chronic lung disease, NYHA functional class, hemoglobin, LA, LVEDD, LVEF, pulmonary hypertension, and severity of VHD entered. Variable importance was evaluated and ranked by the relative weight of predictors, which was defined as the contribution each predictor made to total R^2^.

In survival analyses, variable importance was evaluated and ranked by the proportion of explainable log-likelihood ratio χ^2^ statistics, best subset analysis, as well as the machine learning technique (the random survival forest). The best subset analysis was performed using the “glmulti” package of R software. Feature importance by the random survival forest analysis was investigated and visualized using the “randomForestSRC” and “ggRandomForests” packages of R software. Due to the limited number of events (9/320) in MS, model-specific importance of predictors, which was analyzed by the proportion of explainable log-likelihood ratio χ^2^ statistics, was assessed in the model incorporating age, sex, and the CRH score. Besides, the machine learning method was not applied to obtain feature importance in MS.
